# Sesquiterpenoids and an ergosterol from cultures of the fungus *Daedaleopsis tricolor*

**DOI:** 10.1007/s13659-013-0065-0

**Published:** 2013-11-25

**Authors:** Jiang-Yuan Zhao, Tao Feng, Zheng-Hui Li, Ze-Jun Dong, Hong-Bin Zhang, Ji-Kai Liu

**Affiliations:** 1 State Key Laboratory of Phytochemistry and Plant Resources in West China, Kunming Institute of Botany, Chinese Academy of Sciences, Kunming, 650201 China; 2 Key Laboratory of Medicinal Chemistry for Natural Resources, Ministry of Education, School of Chemical Science and Technology, Yunnan University, Kunming, 650091 China

**Keywords:** *Daedaleopsis tricolor*, sesquiterpenoid, ergosterane

## Abstract

Four new bisabolane sesquiterpenoids daedatrins A-D (**1**–**4**), a cadinane sesquiterpene 12-hydroxy-*α*-cadinol (**5**), and a heptanorergosterane derivative daedatrin G (**6**) were isolated from cultures of the basidiomycete *Daedaleopsis tricolor*. Their structures were elucidated by spectroscopic methods including extensive 2D NMR techniques and X-ray crystallography. All the compounds showed no significant activity against five human cancer cell lines.

**Electronic Supplementary Material:**

Supplementary material is available for this article at 10.1007/s13659-013-0065-0 and is accessible for authorized users.

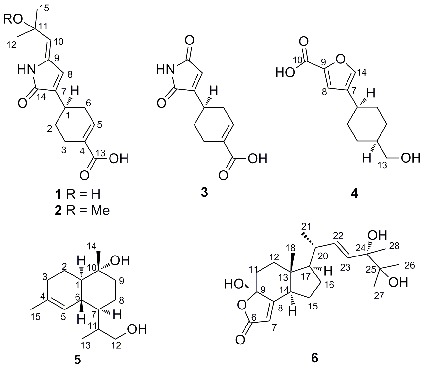

## Introduction

*Daedaleopsis tricolor*, is widely distributed in large areas of China, and is a white wood-rotting fungus.[1] Earlier studies of this fungus resulted in the isolation of fatty acids,[2] terpenes, polysaccharides and other chemical composition.[3] In particular, the triterpenoid 20(29)-lupen-3-one, was isolated from the fruiting body of *D. tricolor*, and also showed strong antifungal, weak antibacterial and antioxidant activities.[4] In order to search for new and active natural products from higher fungi, we investigated the chemical constitutents of cultures of *D. tricolor*, which led to the isolation of six new bisabolane sesquiterpenoids, including three lactams, daedatrins A–C (**1**–**3**), one nor-sesquiterpenoid, daedatrin D (**4**), a cadinane sesquiterpene, 12-hydroxy-*α*-cadinol (**5**), and a heptanorergosterane derivative, daedatrin G (**6**). Their structures have been elucidated on the basis of spectroscopic methods, especially 2D NMR experiments and X-ray crystallography. A noteworthy feature of bisabolane sesquiterpenoids is the preponderance of a six-membered carbon ring,[5] and compound **4** which have been reported in this article is interesting due to the degraded carbons of the side chain. Furthermore, all compounds were evaluated for their cytotoxicities against five human cancer cell lines.

## Results and Discussion

Compound **1** was obtained as colorless crystals (MeOH).

**Figure 1.** Structures of compounds **1**–**6**

The molecular formula $C_{15}H_{19}O_4N$ was determined by HREIMS data at *m/z* 277.1308 (calcd for $C_{15}H_{19}O_4N$, 277.1314), corresponding to seven degrees of unsaturation. The IR data at 3386, 1675 $cm^{-1}$ revealed the presence of hydroxy and carbonyl groups, respectively. The 1D NMR spectra, as well as the HSQC spectrum, revealed 15 carbon resonances, which were ascribed to two methyls, three methylenes, four methines (including three olefinic carbons), six quaternary carbons (including two carbonyl carbons and

*To whom correspondence should be addressed. E-mail: jkliu@mail.kib.ac.cn three olefinic carbons). The $^1$H NMR spectrum showed one amino (NH) resonance at $\delta_H$ 10.14 (1H, s). The $^1$H-$^1$H COSY spectrum analysis provided one spin system (-$CH_2$(3)-$CH_2$(2)-CH(1)-$CH_2$(6)-CH(5)-) as shown in Figure 2. Meanwhile, the proton at $\delta_H$ 7.29 (1H, s, H-5) showed the HMBC correlations to $\delta_C$ 24.7 (C-3) and $\delta_C$ 131.9 (C-4), which suggested that C-1

**Figure 2.** Key 2D NMR correlations of compounds **1**, **3**, and **4**

($\delta_C$ 30.5), C-2 ($\delta_C$ 27.4), C-3, C-4, C-5 ($\delta_C$ 137.8), and C-6 ($\delta_C$ 31.0) were constructed of a six-membered carbon ring A. The correlations from H-3 ($\delta_H$ 2.70) and H-5 to C-13 ($\delta_C$ 169.7) suggested that a carboxyl group was located at C-4. The proton of NH showed significant HMBC correlations to $\delta_C$ 135.9 (C-9) and $\delta_C$ 171.5 (C-14). Furthermore, the proton at $\delta_H$ 6.70 (1H, s, H-8) provided the correlations to $\delta_C$ 140.6 (C-7), C-9, and C-14. These HMBC correlations indicated that an $\gamma$-lactam ring B constructed by C-7, C-8 ($\delta_C$ 131.6), C-9, and C-14 was established. In addition, the HMBC correlations from H-12 ($\delta_H$ 1.56, 3H, s), H-15 ($\delta_H$ 1.56, 3H, s) to C-11 ($\delta_C$ 71.5) and C-10 ($\delta_C$ 121.3), and from H-10 ($\delta_H$ 5.27, 1H, s) to C-8 and C-9 revealed that the existence of an oxygenated isobutyl connected to C-9. All these data suggested that compound **1** was a bisabolane sesquiterpenoid containing an $\gamma$-lactam ring.[5] Fortunately, a single crystal X-ray diffraction experiment not only confirmed the structure of compound **1** but also determined the absolute configuration (Figure 3). Therefore, compound **1** was established to be daedatrin A, as shown.

**Figure 3.** X-ray crystal structure of **1**

Compound **2** was isolated as colorless crystals. It possessed a molecular formula $C_{16}H_{21}O_4N$, according to its HREIMS at $m/z$ 291.1472 ($[M]^+$, calcd 291.1471). The 1D NMR data of **2** (Table 1) were very similar to those of **1** except that a hydroxy in **1** was replaced by one methoxy group ($\delta_C$ 50.4) in **2**, which was supported by the HMBC correlations from $\delta_H$ 3.09 (3H, s, OMe) to $\delta_C$ 71.4/75.7 (s, C-11). Meanwhile, the structure of compound **2** was confirmed by a single crystal X-ray diffraction experiment (Figure 4). Therefore, compound **2** was established to be daedatrin B.

**Figure 4.** X-ray crystal structure of **2**

Compound **3** had the molecular formula $C_{11}H_{11}O_4N$, according to its HREIMS at $m/z$ 221.0691 ($[M]^+$ calcd 221.0688). From the NMR data (Table 2), compound **3** was readily identified as a tetranorterpenoid of **1**. The presence of a carbonyl group located at $\delta_C$ 173.8 (C-9) in compound **3**, which was supported by the HMBC correlations of H-8 ($\delta_H$ 6.35, 1H, d, $J$ = 1.2 Hz) and C-9 (Figure 2). It suggested that the isobutyl (C-10, C-11, C-12, C-15) in **1** was degraded in **3**. The compound has a negative optical rotation in accordance

**Table 1. $^1$H and $^{13}$C NMR spectroscopic data of compounds 1 and 2 ($J$ in Hz)**

| | 1[a] | | 2[a] | |
|---|---|---|---|---|
| pos. | $\delta_C$, type | $\delta_H$, mult. | $\delta_C$, type | $\delta_H$, mult. |
| 1 | 30.5, CH | 2.91, m | 30.5, CH | 2.92, m |
| 2 | 27.4, $CH_2$ | 2.10, dd (12.7, 5.4) | 27.2, CH | 2.10, m |
| | | 1.72, dd (10.5, 5.4) | | 1.72, m |
| 3 | 24.7, $CH_2$ | 2.70, m | 24.7, $CH_2$ | 2.71, m |
| | | 2.52, m | | 2.51, m |
| 4 | 131.9, C | | 132.0/131.9, C | |
| 5 | 137.8, CH | 7.29, s | 137.2/137.7, CH | 7.30, s |
| 6 | 31.0, $CH_2$ | 2.61, m | 31.0, $CH_2$ | 2.61, m |
| | | 2.28, m | | 2.31, m |
| 7 | 140.6, C | | 140.6/140.9, C | |
| 8 | 131.6, CH | 6.70, s | 131.7, CH | 6.69, s |
| 9 | 135.9, C | | 135.9, C | |
| 10 | 121.3, CH | 5.27, s | 121.3/117.2, CH | 5.27/5.00, s |
| 11 | 71.5, C | | 71.4/75.7, C | |
| 12 | 31.4, $CH_3$ | 1.56, s | 31.4, $CH_3$ | 1.56, s |
| 13 | 169.7, C | | 169.7, C | |
| 14 | 171.5, C | | 171.5/172.3, C | |
| 15 | 31.4, $CH_3$ | 1.56, s | 27.4, $CH_3$ | 1.35, s |
| OMe | | | 50.4, $CH_3$ | 3.09, s |
| NH | | 10.14, s | | 10.20/10.49, s |

[a]400 and 100 MHz, in pyridine-$d_5$

with the absolute configuration as **1**. Accordingly, compound **3** was determined to be daedatrin C.

Compound **4** was established to have the molecular formula of $C_{12}H_{16}O_4$ by the HRESIMS at *m/z* 225.1127 $[M + H]^+$ (calcd for $C_{12}H_{17}O_4$, 225.1126). The $^{13}C$ and DEPT NMR spectra (Table 2) exhibited 12 carbon resonances, including five methylenes (one oxygenated), two methines, four olefinic carbons, and a carbonyl carbon. In the $^1H$-$^1H$ COSY spectrum, the correlations established the structural fragment as shown in Figure 2. The HMBC correlations of H-1 ($\delta_H$ 2.46, 1H, m), H-2 ($\delta_H$ 2.03, 1H, m, 1.40, 1H, m), and H-6 ($\delta_H$ 2.03, 1H, m, 1.40, 1H, m) with C-7 ($\delta_C$ 134.8) revealed the connections of C-1 ($\delta_C$ 35.8) to C-7. The HMBC correlations from $\delta_H$ 7.16 (1H, s, H-8), 7.52 (1H, s, H-14) to $\delta_C$ 146.3 (s, C-9) and C-7 indicated the presence of a 2,4-disubstituted furan ring, established by C-7, C-8 ($\delta_C$ 118.7), C-9, C-14 ($\delta_C$ 143.3) and one oxygen atom, In addition, the HMBC correlations from H-8 to $\delta_C$ 162.2 (s, C-10) suggested the existence of a carbonyl group connected to C-9. These data suggested that **4** was a trinor-bisabolane sesquiterpenoid at C-11, C-12, and C-15. Finally, the X-ray diffraction determined the absolute structure of **4** as shown in Figure 5. It was named to be daedatrin D.

**Table 2. $^1H$ and $^{13}C$ NMR spectroscopic data of compounds 3 and 4 (*J* in Hz)**

| | 3[a] | | 4[b] | |
|---|---|---|---|---|
| pos. | $\delta_C$, type | $\delta_H$, mult. | $\delta_C$, type | $\delta_H$, mult. |
| 1 | 31.5, CH | 2.77, m | 35.8, CH | 2.46, m |
| 2 | 27.9, $CH_2$ | 2.07, m; 1.65, m | 34.2, $CH_2$ | 2.03, m; 1.40, m |
| 3 | 24.9, $CH_2$ | 2.43, m; 2.30, m | 30.5, $CH_2$ | 1.93, m; 1.12, m |
| 4 | 131.7, C | | 41.2, CH | 1.52, m |
| 5 | 138.9, CH | 7.02, s | 30.5, $CH_2$ | 1.93, m; 1.12, m |
| 6 | 31.3, $CH_2$ | 2.57, m; 2.26, m | 34.2, $CH_2$ | 2.03, m; 1.40, m |
| 7 | 154.9, C | | 134.8, C | |
| 8 | 127.7, CH | 6.35, d, (1.2) | 118.7, CH | 7.16, s |
| 9 | 173.8, C | | 146.3, C | |
| 10 | | | 162.2, C | |
| 13 | 170.7, C | | 68.6, $CH_2$ | 3.42, d (6.3) |
| 14 | 174.0, C | | 143.3, CH | 7.52, s |

[a]600 and 150 MHz, in methanol-$d_4$;
[b]400 and 100 MHz, in methanol-$d_4$.

The molecular formula of **5** was determined to be $C_{15}H_{26}O_2$ on the basis of a positive-ion HRESIMS at *m/z* 261.1827 $[M + Na]^+$ (calcd for $C_{15}H_{26}O_2Na$, 261.1830). The $^{13}C$ and DEPT NMR spectra (Table 3) exhibited 15 carbon resonances, which consisted of two tertiary methyls, one secondary methyl, five methylenes (one oxygenated), four methines, two olefinic carbons, and an oxygenated quaternary carbon. On the basis of the above evidence, compound **5** was suggested to be a cadinane-type sesquiterpenoid similar to 12,15-dihydroxy-$\alpha$-cadinol.[6] The only difference was the methyl at C-4 ($\delta_C$ 135.6) in **5** rather than a hydroxymethyl in 12,15-dihydroxy-$\alpha$-cadinol. This was confirmed by HMBC correlations of Me-15 ($\delta_H$ 1.64, 3H, s) with C-3 ($\delta_C$ 31.1), C-4, and C-5 ($\delta_C$ 121.9). Thus, the structure of **5** was elucidated as 12-hydroxy-$\alpha$-cadinol.

Compound **6** was obtained as colorless crystals (MeOH), giving the molecular formula $C_{21}H_{32}O_5$ by the HREIMS at *m/z*

**Figure 5.** X-ray crystal structure of **4**

364.2267 $[M]^+$ (calcd 364.2250), corresponding to five degrees of unsaturation. In the $^1H$ NMR spectrum (Table 3), the singlet signals of four tertiary methyl groups and one doublet of secondary methyl group were observed. The $^{13}C$ and DEPT NMR spectra (Table 3) displayed 21 carbon resonances, which were ascribed to five methyls, four methylenes, three methines, a disubstituted donble bond, a trisubstituted donble bond, five quaternary carbon atoms including three oxygenated one, and a carbonyl carbon. The above-mentioned data exhibited similarities with those of phellinignincisterol C.[7] The only difference is the position of the OH group at C-24 ($\delta_C$ 77.8) in **6** rather than at C-28 in phellinignincisterol C, as confirmed by HMBC correlations of Me-26 ($\delta_H$ 1.18, 3H, s), Me-27 ($\delta_H$ 1.16, 3H, s), and Me-28 ($\delta_H$ 1.25, 3H, s) with C-24. The X-ray diffraction not only confirmed the structure of compound **6** as elucidated above but also determined the absolute configuration (Figure 6). Therefore, compound **6** was established to be daedatrin G.

**Figure 6.** X-ray crystal structure of **6**

Compounds **1**–**6** were tested for cytotoxicities against five human cancer cell lines, MCF-7 breast, SMMC-7721 hepatocellular carcinoma, HL-60 myeloid leukemia, SW480 colon cancer, and A-549 lung cancer. Unfortunately, no significant activity was detected ($IC_{50} > 40\ \mu M$).

## Experimental Section

**General Experimental Procedures.** Melting points were obtained on a Yuhua X-4 digital microdisplaying melting point apparatus. Optical rotations were recorded on a Horiba SEPA-

**Table 3. $^1$H and $^{13}$C NMR spectroscopic data of compounds 5 and 6 (*J* in Hz)**

| | 5[a] | | 6[b] | |
|---|---|---|---|---|
| pos. | $\delta_C$, type | $\delta_H$, mult. | $\delta_C$, type | $\delta_H$, mult. |
| 1 | 50.2, CH | 1.23, m | | |
| 2 | 22.8, $CH_2$ | 1.22, m; 1.99, m | | |
| 3 | 31.1, $CH_2$ | 1.97, m; 1.99, m | | |
| 4 | 135.6, C | | | |
| 5 | 121.9, CH | 5.44, m | | |
| 6 | 39.4, CH | 1.76, m | 173.6, C | |
| 7 | 41.9, CH | 1.32, m | 112.7, CH | 5.68, d (1.7) |
| 8 | 22.6, $CH_2$ | 1.47, m; 2.06, m | 173.5, C | |
| 9 | 42.1, $CH_2$ | 1.44, m; 1.78, m | 107.3, C | |
| 10 | 72.5, C | | | |
| 11 | 34.7, CH | 2.16, m | 36.1, $CH_2$ | 1.79, m; 2.24, m |
| 12 | 67.3, $CH_2$ | 3.49, dd (10.5, 7.3); 3.52, dd (10.5, 7.3) | 36.4, $CH_2$ | 1.62, m; 1.98, m |
| 13 | 10.4, $CH_3$ | 0.79, d (7.0) | 49.9, C | |
| 14 | 20.9, $CH_3$ | 1.09, s | 51.7, CH | 2.66, m |
| 15 | 24.1, $CH_3$ | 1.64, s | 22.3, $CH_2$ | 1.61, m; 1.72, m |
| 16 | | | 30.2, $CH_2$ | 1.50, m; 1.93, m |
| 17 | | | 56.7, CH | 1.55, m |
| 18 | | | 12.1, $CH_3$ | 0.66, s |
| 19 | | | | |
| 20 | | | 41.5, CH | 2.15, m |
| 21 | | | 21.1, $CH_3$ | 1.08, s |
| 22 | | | 135.8, CH | 5.54, dd (15.3, 7.3) |
| 23 | | | 133.6, CH | 5.64, dd (15.3) |
| 24 | | | 77.8, C | |
| 25 | | | 75.7, C | |
| 26 | | | 25.3, $CH_3$ | 1.18, s |
| 27 | | | 25.1, $CH_3$ | 1.16, s |
| 28 | | | 23.0, $CH_3$ | 1.25, s |

[a]600 and 150 MHz, in $CDCl_3$; [b]400 and 100 MHz, in methanol-$d_4$.

300 polarimeter. UV spectra were obtained on a Shimadzu UV-2401A spectrophotometer. A Tenor 27 spectrophotometer was used for IR spectroscopy using KBr pellets. NMR spectra were obtained on Avance III 600, Bruker DRX-500, and Bruker AM-400 spectrometers with Tetramethylsilane (TMS) used as an internal standard at room temperature. Chemical shifts ($\delta$) were expressed in ppm with reference to the solvent signals. HRESIMS and HREIMS were recorded on an API-Qstar-Pulsar-1 spectrometer. X-ray crystallographic data were collected on a Bruker APEX DUO instrument. Column chromatography (CC) was performed on Silica gel (200–300 mesh, Qingdao Haiyang Chemical Co. Ltd., Qingdao, China), Sephadex LH-20 (Pharmacia, Piscataway, NJ, USA). MPLC was performed on a BUCHI Sepacore system (BUCHI Labortechnik AG, Switzerland), and columns packed with RP-18 (40–75 $\mu$m, Fuji Silysia Chemical Ltd., Japan). An Agilent 1100 series instrument equipped with Agilent ZORBAX SB-C18 column (5 $\mu$m, 4.6 mm × 150 mm) was used for HPLC analysis, and a semi-preparative Agilent ZORBAX SB-C18 column (5 $\mu$m, 9.4 mm × 150 mm) was used for the sample preparation. Fractions were monitored using TLC (GF 254, Qingdao Haiyang Chemical Co. Ltd.), and spots were visualized by 10% $H_2SO_4$ in ethanol.

**Fungus Material and Cultivation Conditions.** The fungus *Daedaleopsis tricolor* was collected from Changbai Mountain, Jilin Province, China, in August 2010. The fungus was identified by Prof. Tolgor at Jilin Agricultural University. A voucher specimen is deposited at State Key Laboratory of Phytochemistry and Plant Resources in West China, Kunming Institute of Botany, Chinese Academy of Sciences. The mycelial cultures were derived from tissue plugs. The culture medium consisted of glucose (5%), peptone from porcine meat (0.15%), yeast powder (0.5%), $KH_2PO_4$ (0.05%) and $MgSO_4$ (0.05%). Inoculums of *D. tricolor* were prepared in a 15 L-fermenter (Biostar, Shanghai GuoQiang, China) for 6 days under the following conditions: culture temperature 24 °C, initial pH 6.0, agitation speed 250 rpm, inoculation volume 10% (by volume), and aeration rate 1.0 vvm. Then, the liquid seed was transferred into a 100 L-fermentation tank to be cultivated under the same conditions for 20 days to produce a 80 L culture broth.

**Extraction and Isolation.** The culture broth (80 L) of *Daedaleopsis tricolor* was filtered, and the filtrate was extracted three times with EtOAc, while the mycelium was extracted three times with $CHCl_3$/MeOH (1:1). The EtOAc layer together with the mycelium extraction was concentrated under reduced pressure to give a crude extract (130 g). The extract was subjected to column chromatography over silica gel (200–300 mesh) eluted with a gradient of $CHCl_3$/MeOH (1:0 → 0:1) to obtain 9 fractions (1–9). Fraction 5 (23 g) was applied to MPLC ($MeOH/H_2O$, eluting from 2:8 to 10:0 for 120 minutes with a flow rate of 20 mL/min) to give 12 subfractions (A–L). Subfraction F (2.1 g) was separated using a Sephadex LH-20 (MeOH) column chromatography and on a semi-preparative HPLC ($MeCN/H_2O$, 20/80) to elute **1** (14 mg) and **2** (23 mg). Subfraction H (1.2 g) was isolated and purified repeatedly by Sephadex LH-20 (MeOH) column chromatography, silica gel column chromatography (petroleum ether/$Me_2CO$, 3:1), then on Sephadex LH-20 ($Me_2CO$) column chromatography to give **3** (3.1 mg). Subfraction G (1.8 g) was separated by Sephadex LH-20 (MeOH) column chromatography, silica gel column chromatography (petroleum ether/$Me_2CO$, 5:1 → 2:1), then on Sephadex LH-20 ($Me_2CO$) column chromatography to give **4** (7.6 mg). Subfraction K (2.2 g) was separated by silica gel column chromatography (petroleum ether/$Me_2CO$, 7:1 → 1:1), then on semi-preparative HPLC ($MeCN/H_2O$, 32/68) to give **6** (2.9 mg). Fraction 8 (4.3 g) was applied to silica gel column, eluted with petroleum ether/$Me_2CO$ (2:1 → 1:1) to give 4 subfractions (A–D). Subfraction C (120 mg) was separated by Sephadex LH-20 (MeOH) column chromatography and then on semi-preparative HPLC ($MeCN/H_2O$, 40/60) to give **5** (1.5 mg).

**Daedatrin A (1):** colorless crystals (MeOH); mp 195–197 °C; $[\alpha]^{26.0}_{D}$ – 119.9 (*c* 0.27, MeOH). UV (MeOH) $\lambda_{max}$ (log $\varepsilon$) 278 (4.40), 203 (4.18) nm; IR (KBr) $\nu_{max}$ 3386, 2926, 2855, 1675, 1635, 1423, 1362, 1286, 868, 709 $cm^{-1}$; $^1H$ (400 MHz, pyridine-$d_5$) and $^{13}C$ NMR (100 MHz, pyridine-$d_5$) spectroscopic data see Table 1; HREIMS *m/z* 277.1308 $[M]^+$ (calcd for $C_{15}H_{19}O_4N$, 277.1314).

**Daedatrin B (2):** colorless crystals (MeOH); mp 192–194 °C; $[\alpha]^{21.8}_{D}$ – 112.5 (*c* 0.13, MeOH). UV (MeOH) $\lambda_{max}$ (log $\varepsilon$) 278 (4.34), 203 (4.09) nm; IR (KBr) $\nu_{max}$ 3428, 3302, 2925, 1680, 1639, 1424, 1273, 1058 $cm^{-1}$; $^1H$ (400 MHz, pyridine-$d_5$) and $^{13}C$ NMR (100 MHz, pyridine-$d_5$) spectroscopic data see Table 1; HREIMS *m/z* 291.1472 $[M]^+$ (calcd for $C_{16}H_{21}O_4N$, 291.1471).

**Daedatrin C (3):** white, amorphous powder; $[\alpha]^{24.6}_{D}$ – 24.8 (*c* 0.08, MeOH). UV (MeOH) $\lambda_{max}$ (log $\varepsilon$) 219 (2.86), 202 (2.83) nm; IR (KBr) $\nu_{max}$ 3430, 2924, 1712, 1630, 1384, 1118 $cm^{-1}$; $^1H$ (600MHz, methanol-$d_4$) and $^{13}C$ NMR (150 MHz, methanol-$d_4$) spectral data see Table 2; HREIMS *m/z* 221.0691 $[M]^+$ (calcd for $C_{11}H_{11}O_4N$, 221.0688).

**Daedatrin D (4):** colorless crystals (MeOH); mp 179–181 °C; $[\alpha]^{19.7}_{D}$ – 11.1 (*c* 0.10, MeOH). UV (MeOH) $\lambda_{max}$ (log $\varepsilon$) 253 (3.47), 218 (3.17), 198 (3.19) nm; IR (KBr) $\nu_{max}$ 3423, 2923, 2852, 1682, 1628, 1591, 1403, 1324, 1282, 1251, 1191, 1116, 1084, 967, 943 $cm^{-1}$; $^1H$ (400 MHz, methanol-$d_4$) and $^{13}C$ NMR (100 MHz, methanol-$d_4$) spectral data see Table 2; Positive HRESIMS *m/z* 225.1127 $[M + H]^+$ (calcd for $C_{12}H_{17}O_4$, 225.1126).

**12-Hydroxy-α-cadinol (5):** colorless oil; $[\alpha]^{12.6}_{D}$ + 43.2 (*c* 0.10, MeOH). UV (MeOH) $\lambda_{max}$ (log $\varepsilon$) 227 (3.49), 202 (3.87) nm; IR (KBr) $\nu_{max}$ 3430, 2923, 2871, 1713, 1631, 1455, 1381, 1122, 1028, 576 $cm^{-1}$; $^1H$ (600 MHz, $CDCl_3$) and $^{13}C$ NMR (150 MHz, $CDCl_3$) spectroscopic data see Table 3; Positive HRESIMS *m/z* 261.1827 $[M + Na]^+$ (calcd for $C_{15}H_{26}O_2Na$, 261.1830).

**Daedatrin G (6):** colorless crystals (MeOH); mp 170–172 °C; $[\alpha]^{21.3}_{D}$ + 36.1 (*c* 0.11, MeOH). UV (MeOH) $\lambda_{max}$ (log $\varepsilon$) 216 (3.62) nm; IR (KBr) $\nu_{max}$ 3441, 1628, 1413, 1384, 1272, 1221, 1172, 1109, 1065, 581 $cm^{-1}$; $^1H$ (400 MHz, methanol-$d_4$) and $^{13}C$ NMR (100 MHz, methanol-$d_4$) spectroscopic data see Table 3; Positive HREIMS *m/z* 364.2267 $[M]^+$ (calcd for $C_{21}H_{32}O_5$, 364.2250).

**X-ray crystallographic analysis of compound 1:** Colorless blocks, $C_{15}H_{19}NO_4$, $M$ = 277.31, triclinic, space group $P1$, $a$ = 7.7149(2) Å, $b$ = 9.7181(3) Å, $c$ = 9.7739(3) Å, $\alpha$ = 95.4840(10)°, $\beta$ = 91.2320(10)°, $\gamma$ = 108.2790(10)°, $V$ = 691.60(4) $Å^3$, $Z$ = 2, $d_x$ = 1.332 $Mg/m^3$, $F(000)$ = 296, $\mu$(Cu K$\alpha$) = 0.795 $mm^{-1}$, crystal dimensions 0.55 × 0.30 × 0.10 $mm^3$ was used for measurement on a Bruker APEX DUO with a graphite monochromater, Cu K$\alpha$ radiation. The total number of reflections measured was 8954, of which 2366 were observed, $I > 2\sigma(I)$. Final indices: $R_1$ = 0.0946, w$R_2$ = 0.2532 $S$ = 1.177. The crystal structure of **1** was solved by direct method SHLXS-97 and expanded using difference Fourier technique,[8] refined by the program SHLXL-97 and the full-matrix least-squares calculations. The Flack[9] parameter = 0.03(6), indicating that the absolute structure has been determined correctly.

**X-ray crystallographic analysis of compound 2:** Colorless blocks, 2($C_{16}H_{21}NO_4$), $M$ = 582.68, monoclinic, space group $P21$, $a$ = 6.50440(10) Å, $b$ = 18.0530(3) Å, $c$ = 13.0427(2) Å, $\alpha$ = 90.00°, $\beta$ = 100.9960(10)°, $\gamma$ = 90.00°, $V$ = 1503.41(4) $Å^3$, $Z$ = 2, $d_x$ = 1.287 $Mg/m^3$, $F(000)$ = 624, $\mu$(Cu K$\alpha$) = 0.756 $mm^{-1}$, crystal dimensions 0.32 × 0.14 × 0.13 $mm^3$ was used for measurement on a Bruker APEX DUO with a graphite monochromater, Cu K$\alpha$ radiation. The total number of reflections measured was 22349, of which 5367 were observed, $I > 2\sigma(I)$. Final indices: $R_1$ = 0.0408, w$R_2$ = 0.1099 $S$ = 1.046. The crystal structure of **2** was solved by direct method SHLXS-97 and expanded using difference Fourier technique, refined by the program SHLXL-97 and the full-matrix least-squares calculations. The Flack parameter = -0.03(15), indicating that the absolute structure has been determined correctly.

**X-ray crystallographic analysis of compound 4:** Colorless blocks, $C_{12}H_{16}O_4$, $M$ = 224.25, monoclinic, space group $P21/n$, $a$ = 11.1726(2) Å, $b$ = 6.06790(10) Å, $c$ = 15.7985(3) Å, $\alpha$ = 90.00°, $\beta$ = 91.4050(10)°, $\gamma$ = 90.00°, $V$ = 1070.72(3) $Å^3$, $Z$ = 4, $d_x$ = 1.391$Mg/m^3$, $F(000)$ = 480, $\mu$(Cu K$\alpha$) = 0.861 $mm^{-1}$, crystal dimensions 1.30 × 0.45 × 0.15 $mm^3$ was used for measurement on a Bruker APEX DUO with a graphite monochromater, Cu K$\alpha$ radiation. The total number of reflections measured was 8232, of which 1892 were observed, $I > 2\sigma(I)$. Final indices: $R_1$ = 0.0488, w$R_2$ = 0.1396 $S$ = 1.134. The crystal structure of **4** was determined by direct method SHLXS-97 and expanded using difference Fourier technique, refined by the program SHLXL-97 and the full-matrix least-squares calculations. The Flack parameter = 0.02(2), indicating that the absolute structure has been determined correctly.

**X-ray crystallographic analysis of compound 6:** Colorless blocks, $2(C_{21}H_{32}O_5)\cdot3(H_2O)$, $M$ = 782.98, monoclinic, space group $P21$, $a$ = 12.6179(3) Å, $b$ = 6.33140(10) Å, $c$ = 26.5136(6) Å, $\alpha$ = 90.00°, $\beta$ = 101.6110(10)°, $\gamma$ = 90.00°, $V$ = 2074.80(8) Å$^3$, $Z$ = 2, $d_x$ = 1.253Mg/m$^3$, $F(000)$ = 852, $\mu$(Cu Kα) = 0.749 mm$^{-1}$, crystal dimensions 0.43 × 0.05 × 0.05 mm$^3$ was used for measurement on a Bruker APEX DUO with a graphite monochromater, Cu K$\alpha$ radiation. The total number of reflections measured was 16345, of which 6762 were observed, $I > 2\sigma(I)$. Final indices: $R_1$ = 0.0503, w$R_2$ = 0.1267 $S$ = 1.064. The crystal structure of **6** was determined using a direct method SHLXS-97 and expanded using difference Fourier technique, refined by the program SHLXL-97 and the full-matrix least-squares calculations. The Flack parameter = –0.15(12), indicating that the absolute structure has been determined correctly.

Crystallographic data for these structures have been deposited with the Cambridge Crystallographic Data Center as CCDC 912939 for **1**, CCDC 912940 for **2**, CCDC 912938 for **4** and CCDC 912941 for **6**. Copies of the data can be obtained free of charge on application to CCDC via the Internet at www.ccdc.com.ac.uk/conts/retrieving.html (or from the Cambridge Crystallographic Data Centre 12, Union Road, Cambridge CB2 1EZ, UK, fax: +44 1223-336-033; e-mail: deposit@ccdc.cam.ac.uk).

**Cytotoxicity Assay.** Five human cancer cell lines, breast cancer MCF-7, hepatocellular carcinoma SMMC-7721, human myeloid leukemia HL-60, colon cancer SW480, and lung cancer A-549 cells, were used in the cytotoxic assay. All the cells were cultured in RPMI-1460 or DMEM medium (Hyclone, USA), supplemented with 10% fetal bovine serum (Hyclone, USA) in 5% $CO_2$ at 37 °C. The cytotoxicity assay was performed according to the MTT (3-(4,5-dimethylthiazol-2-yl)-2,5-diphennyl tetrazolium bromide) method in 96-well microplates.[10] Briefly, 100 $\mu$L adherent cells were seeded into each well of 96-well cell culture plates and allowed to adhere for 12 h before drug addition, while suspended cells were seeded just before drug addition with initial density of 1 × 10$^5$ cells/mL. Each tumor cell line was exposed to the test compound dissolved in DMSO at concentrations of 0.064, 0.32, 1.6, 8, 40 $\mu$mol in triplicates for 48 h, with cisplatin (Sigma, USA) and taxol (National Institute for the Control of Pharmaceutical and Biological Products, China) as positive controls. After compound treatment, cell viability was detected and a cell growth curve was graphed. $IC_{50}$ values were calculated by Reed and Muench's method.[11]

### Electronic Supplementary Material

Supplementary material is available in the online version of this article at http://dx.doi.org/10.1007/s13659-013-0065-0 and is accessible for authorized users.

### Acknowledgments

This work was financially supported by the National Natural Science Foundation of China (U1132607).

**Open Access** This article is distributed under the terms of the Creative Commons Attribution License which permits any use, distribution, and reproduction in any medium, provided the original author(s) and source are credited.

## Electronic supplementary material

Supplementary material, approximately 5.14 MB.
