# Supplementary material for: Sesquiterpenoids and an ergosterol from cultures of the fungus Daedaleopsis tricolor
Source: Nat Prod Bioprospect. 2013 Nov 25;3(6):271–6. doi: 10.1007/s13659-013-0065-0 (PMC4131599; doi:10.1007/s13659-013-0065-0)
Supplement: Supplementary file 1 — Supplementary material, approximately 5.14 MB. [file 13659_2013_65_MOESM1_ESM.pdf]

Electronic Supplementary Material

Sesquiterpenoids and an ergosterol from cultures of the fungus *Daedaleopsis tricolor*

Jiang-Yuan ZHAO,[a,b] Tao FENG,[b] Zheng-Hui LI,[a] Ze-Jun DONG,[a] Hong-Bin ZHANG,[b] and Ji-Kai LIU[a,]*

[a]State Key Laboratory of Phytochemistry and Plant Resources in West China, Kunming Institute of Botany, Chinese Academy of Sciences, Kunming 650201, China

[b]Key Laboratory of Medicinal Chemistry for Natural Resources, Ministry of Education, School of Chemical Science and Technology, Yunnan University, Kunming 650091, China

Received 21 August 2013; Accepted 10 November 2013

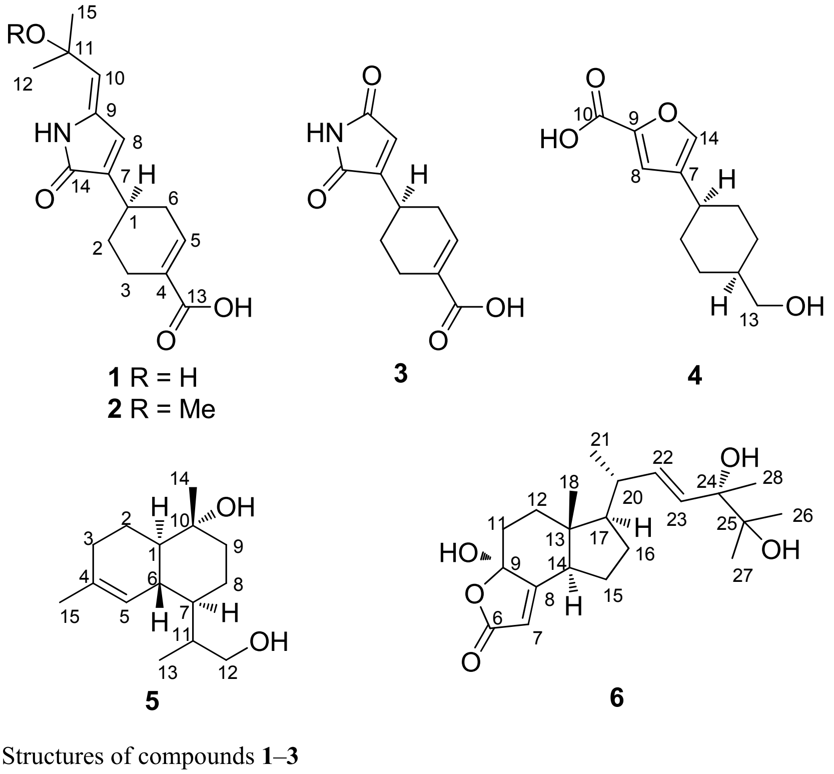

Structures of compounds **1**–**3**

S2-S6 Figures 1-10 NMR, MS, IR, UV spectra and OR of **1**.
S7-S11 Figures 11-20 NMR, MS, IR, UV spectra and OR of **2**.
S12-S16 Figures 21-30 NMR, MS, IR, UV spectra and OR of **3**.
S17-S21 Figures 31-40 NMR, MS, IR, UV spectra and OR of **4**.
S22-S26 Figures 41-50 NMR, MS, IR, UV spectra and OR of **5**.
S27-S31 Figures 51-60 NMR, MS, IR, UV spectra and OR of **6**.

*To whom correspondence should be addressed. E-mail: jkliu@mail.kib.ac.cn

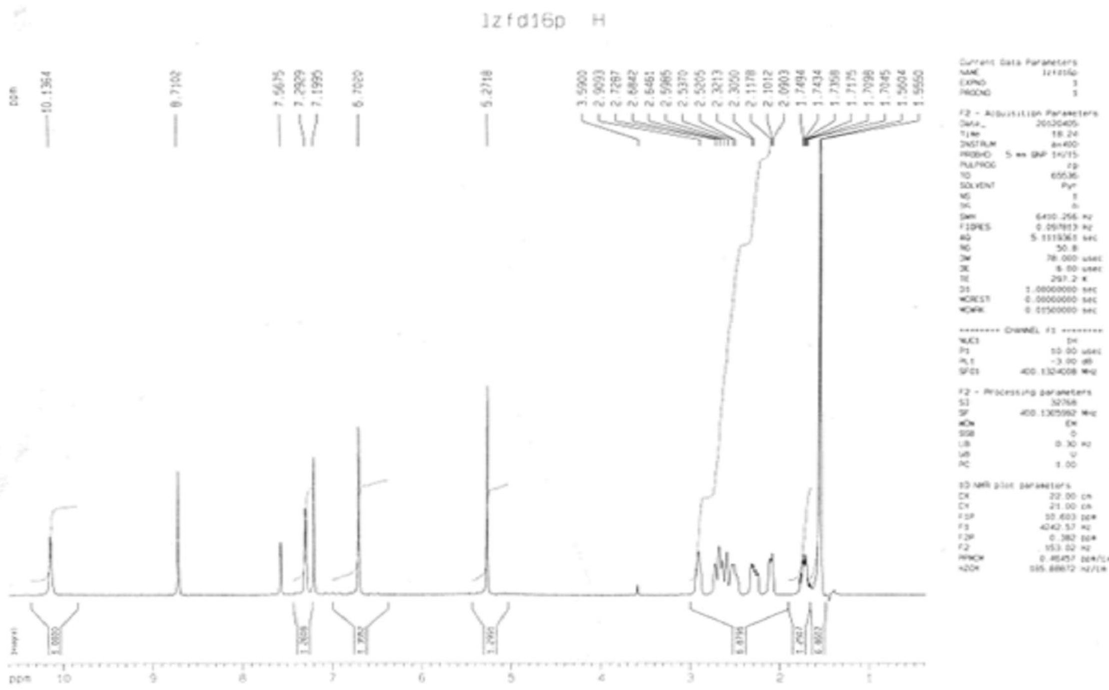

Figure 1. ^{1}H NMR spectrum of **1** (400 MHz, pyridine-d_5).

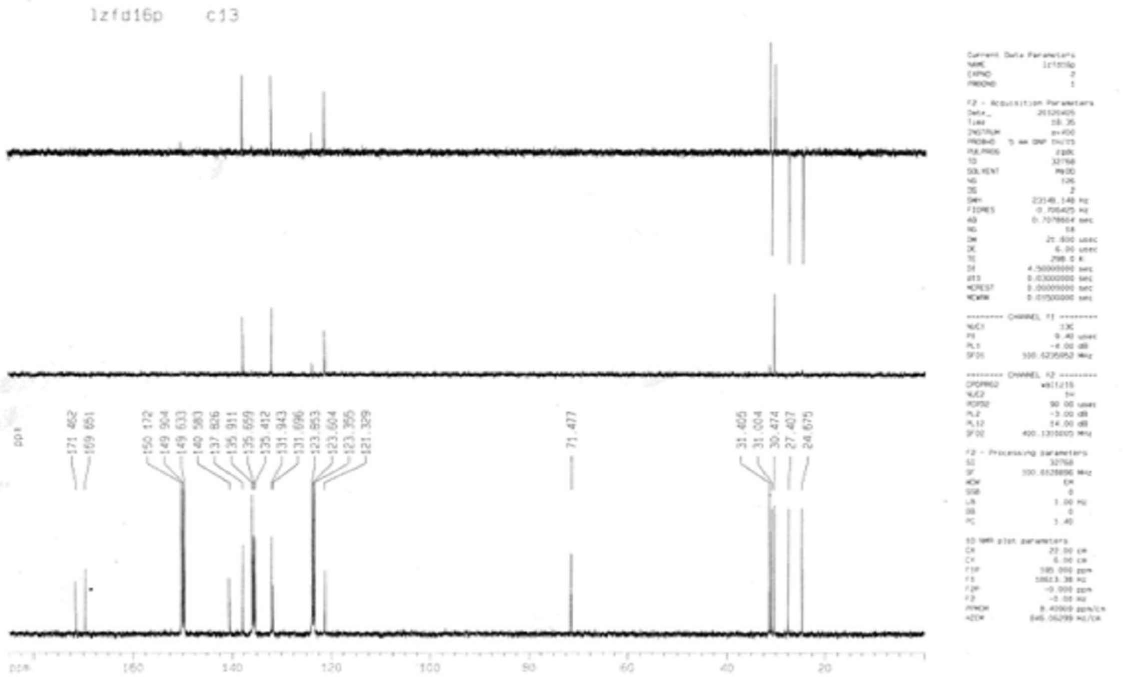

Figure 2. ^{13}C NMR spectrum of **1** (100 MHz, pyridine-d_5).

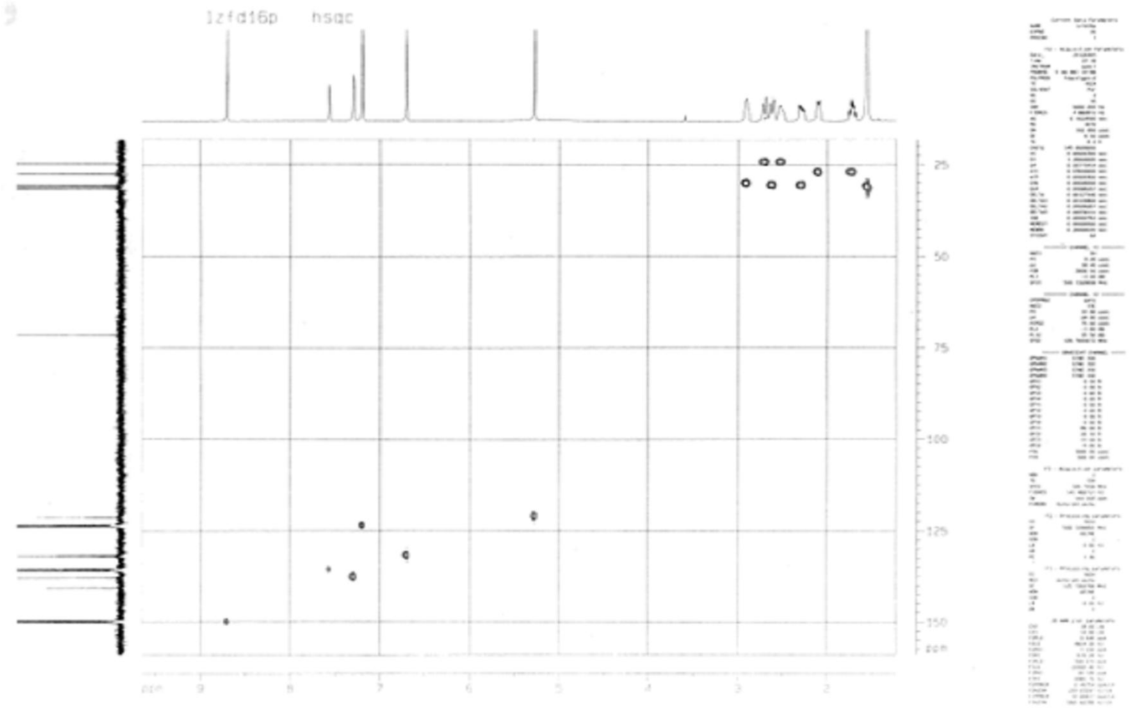

Figure 3. HSQC spectrum of **1** (500 MHz, pyridine-d_5).

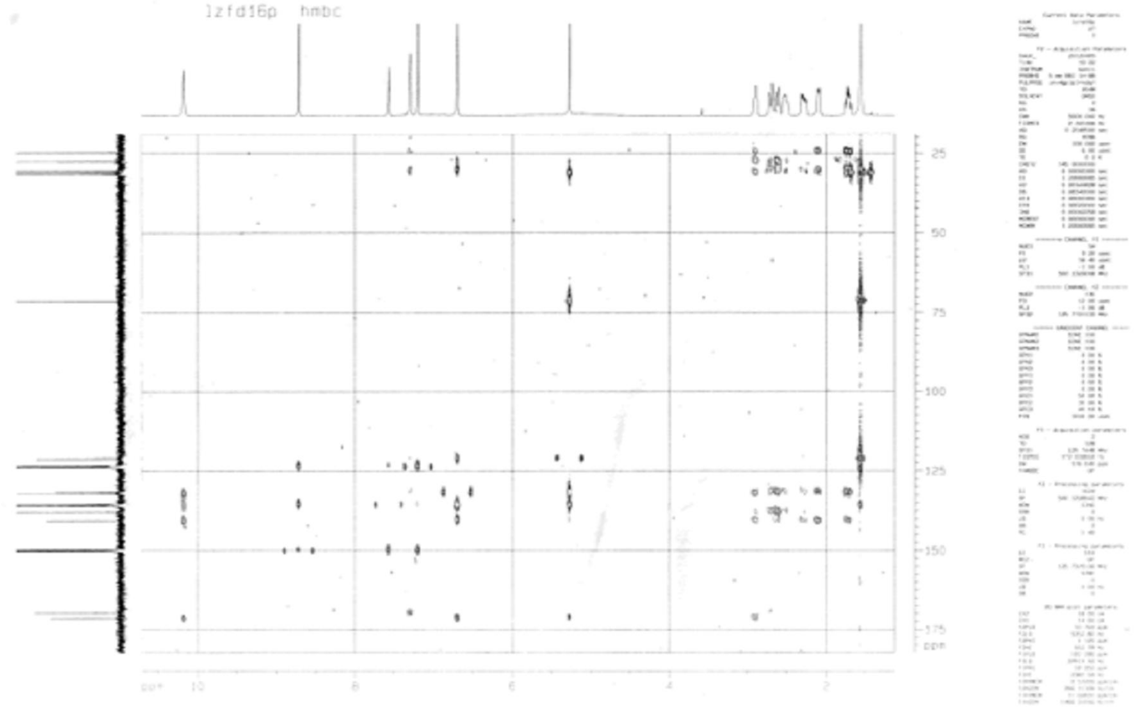

Figure 4. HMBC spectrum of **1** (500 MHz, pyridine-d_5).

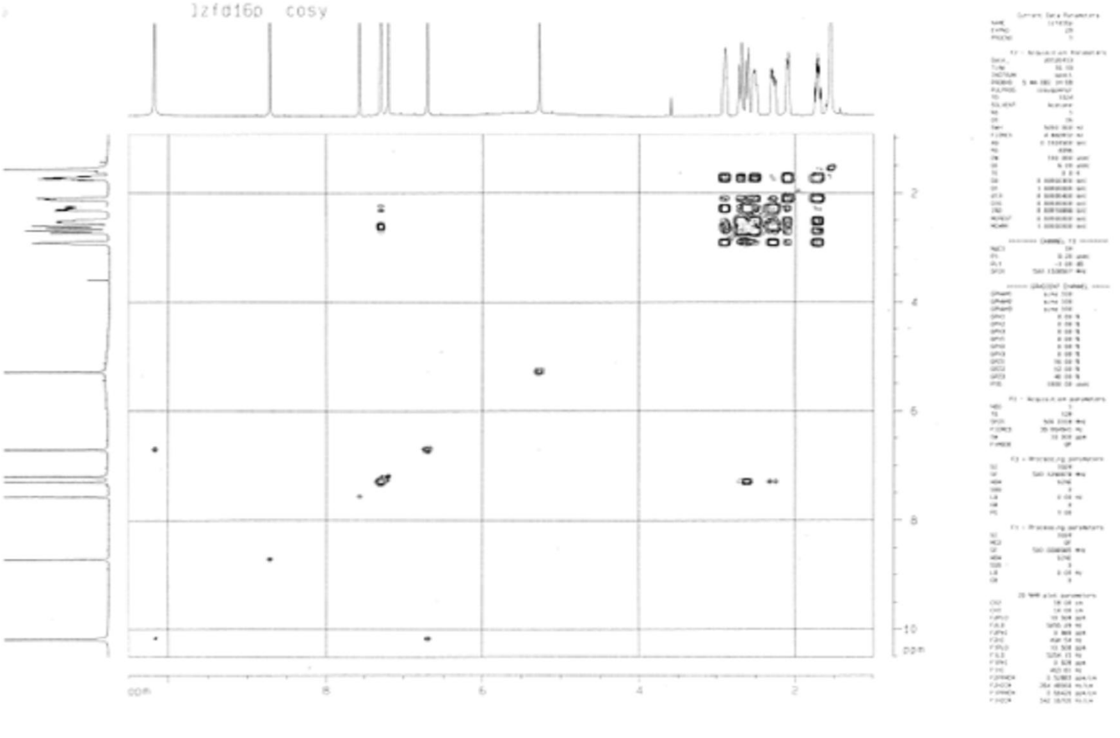

Figure 5. COSY spectrum of **1** (500 MHz, pyridine-d_5).

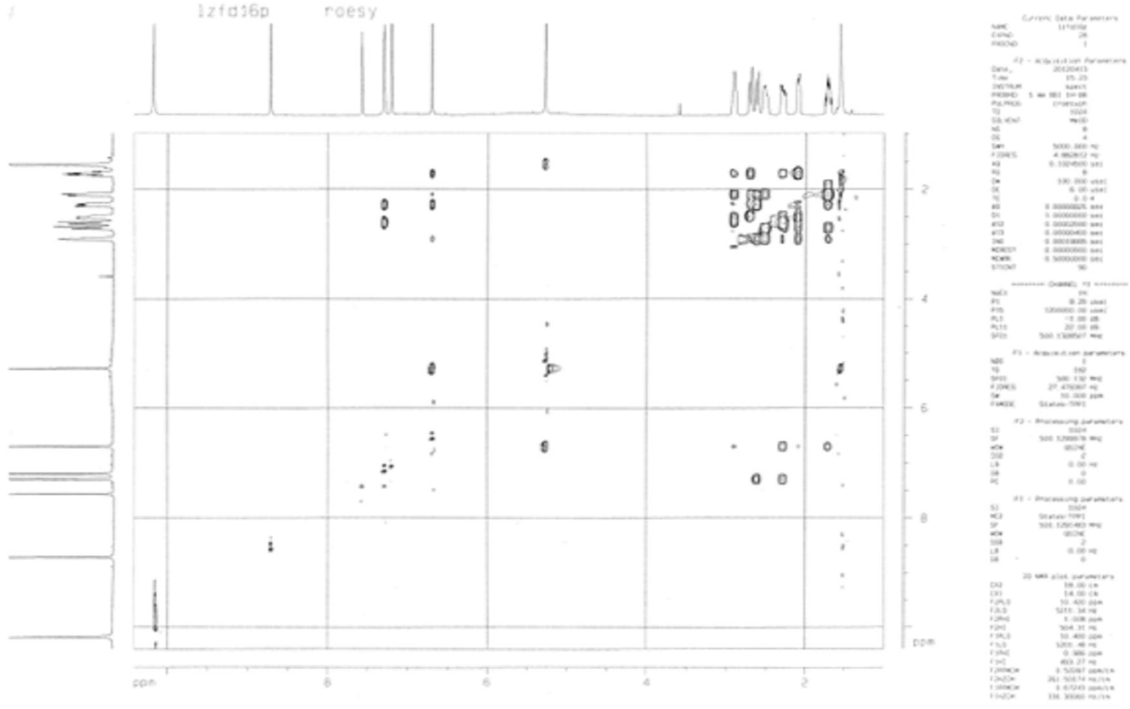

Figure 6. ROESY spectrum of **1** (500 MHz, pyridine-d_5).

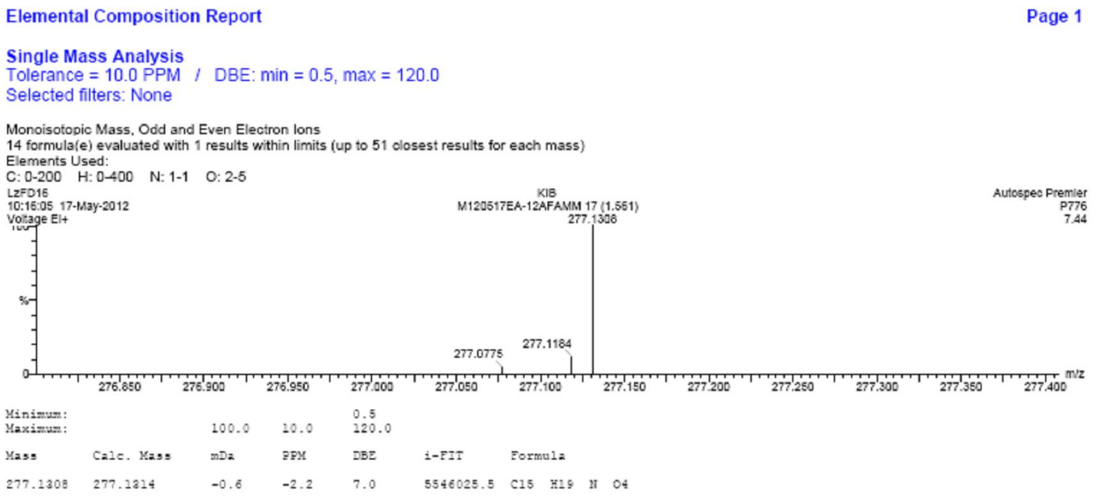

Elemental Composition Report Page 1

Single Mass Analysis
Tolerance = 10.0 PPM / DBE: min = 0.5, max = 120.0
Selected filters: None

Monoisotopic Mass, Odd and Even Electron Ions
14 formula(e) evaluated with 1 results within limits (up to 51 closest results for each mass)
Elements Used:
C: 0-200 H: 0-400 N: 1-1 O: 2-5

Mass	Calc. Mass	mDa	PPM	DBE	i-FIT	Formula
Minimum:				0.5		
Maximum:		100.0	10.0	120.0		
277.1308	277.1314	-0.6	-2.2	7.0	5546025.5	C15 H19 N O4

Figure 7. HR-EI-MS of **1**.

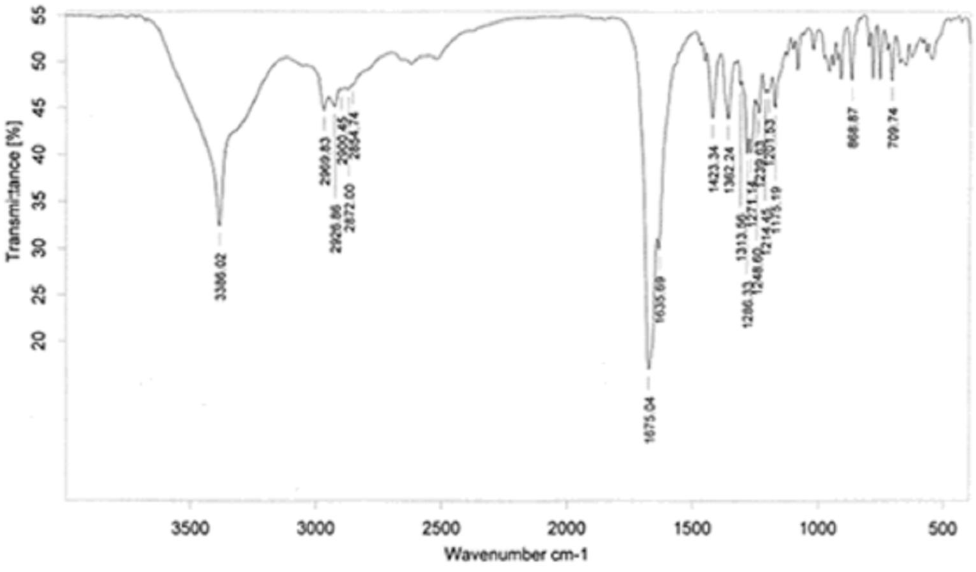

Sample : LzFD16	Frequency Range : 399.246 - 3996.32	Measured on : 17/05/2012	
Technique : KBr压片	Resolution : 4	Instrument : Tensor27	Sample Scans : 16
Customer : 120517IR1	Zerofilling : 2	Acquisition : Double Sided,For	

Figure 8. IR spectrum of **1**.

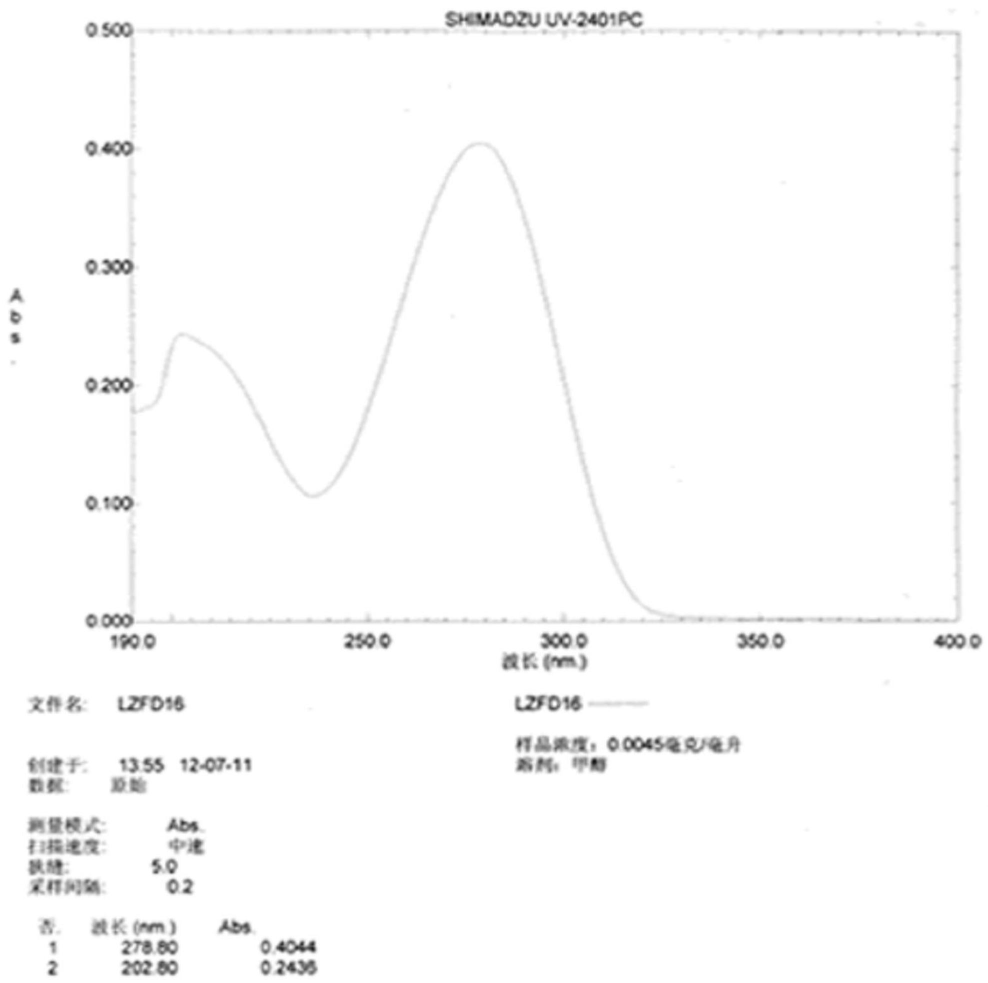

Figure 9. UV spectrum of **1**.

Optical rotation measurement

Model : P-1020 (A060460638)

No.	Sample	Mode	Data	Monitor<br>Blank	Temp.<br>Cell<br>Temp Point	Date<br>Comment<br>Sample Name	Light<br>Filter<br>Operator	Cycle Time<br>Integ Time
No.1	18 (1/3)	Sp.Rot	-118.5190	-0.1600<br>0.0000	26.0<br>50.00<br>Cell	Wed Jul 11 11:42:03 2012<br>0.00270g/mlMeOH<br>LZFD16	Na<br>589nm	2 sec<br>10 sec
No.2	18 (2/3)	Sp.Rot	-120.7410	-0.1630<br>0.0000	26.1<br>50.00<br>Cell	Wed Jul 11 11:42:17 2012<br>0.00270g/mlMeOH<br>LZFD16	Na<br>589nm	2 sec<br>10 sec
No.3	18 (3/3)	Sp.Rot	-120.3700	-0.1625<br>0.0000	26.0<br>50.00<br>Cell	Wed Jul 11 11:42:30 2012<br>0.00270g/mlMeOH<br>LZFD16	Na<br>589nm	2 sec<br>10 sec

-119.8765°

Figure 10. Optical rotation measurement of **1**.

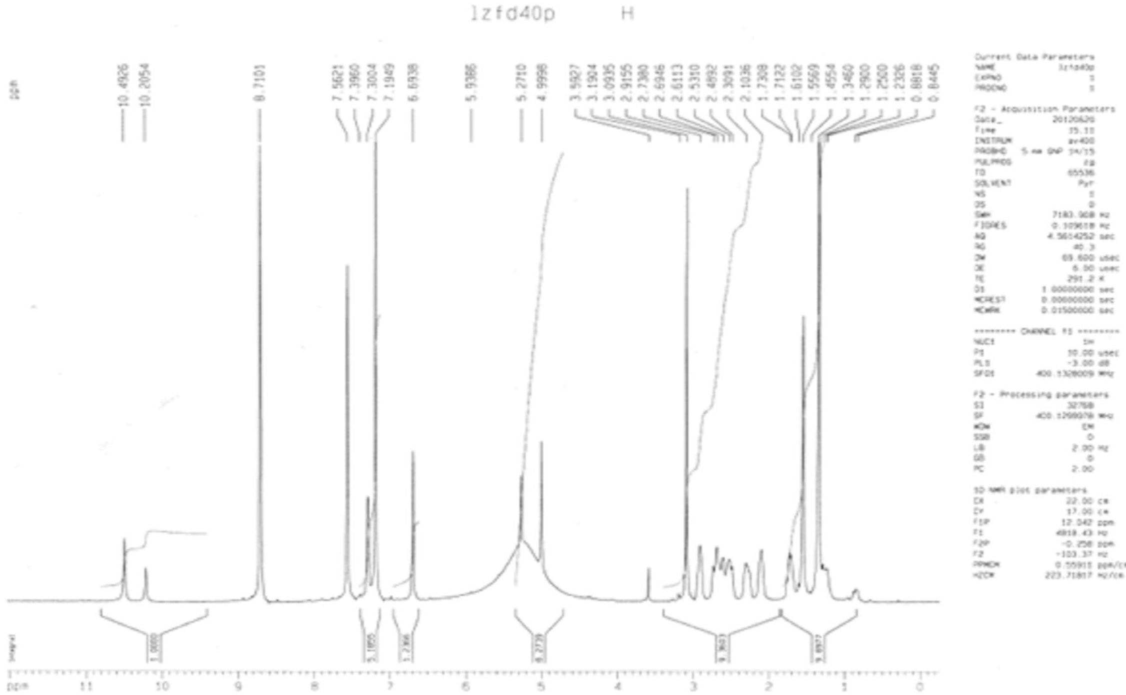

Figure 11. ^{1}H NMR spectrum of **2** (400 MHz, pyridine-d_5).

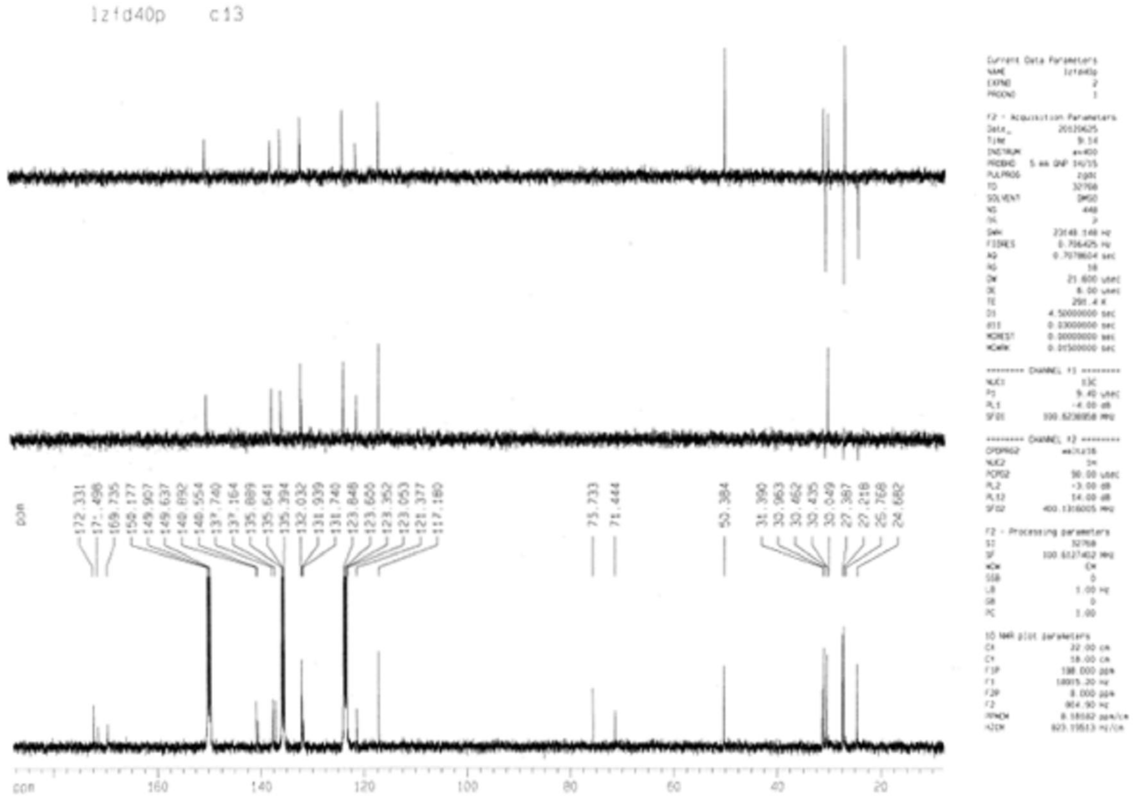

Figure 12. ^{13}C NMR spectrum of **2** (100 MHz, pyridine-d_5).

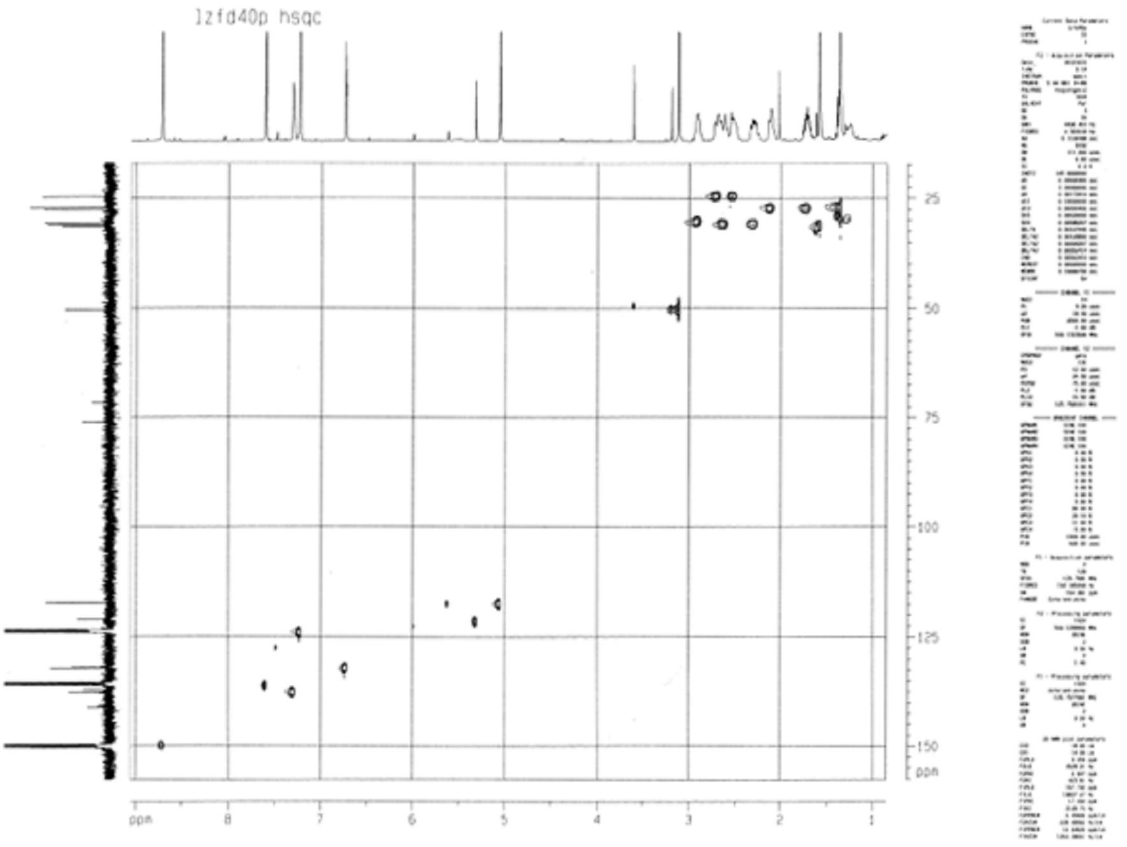

Figure 13. HSQC spectrum of **2** (500 MHz, pyridine-d_5).

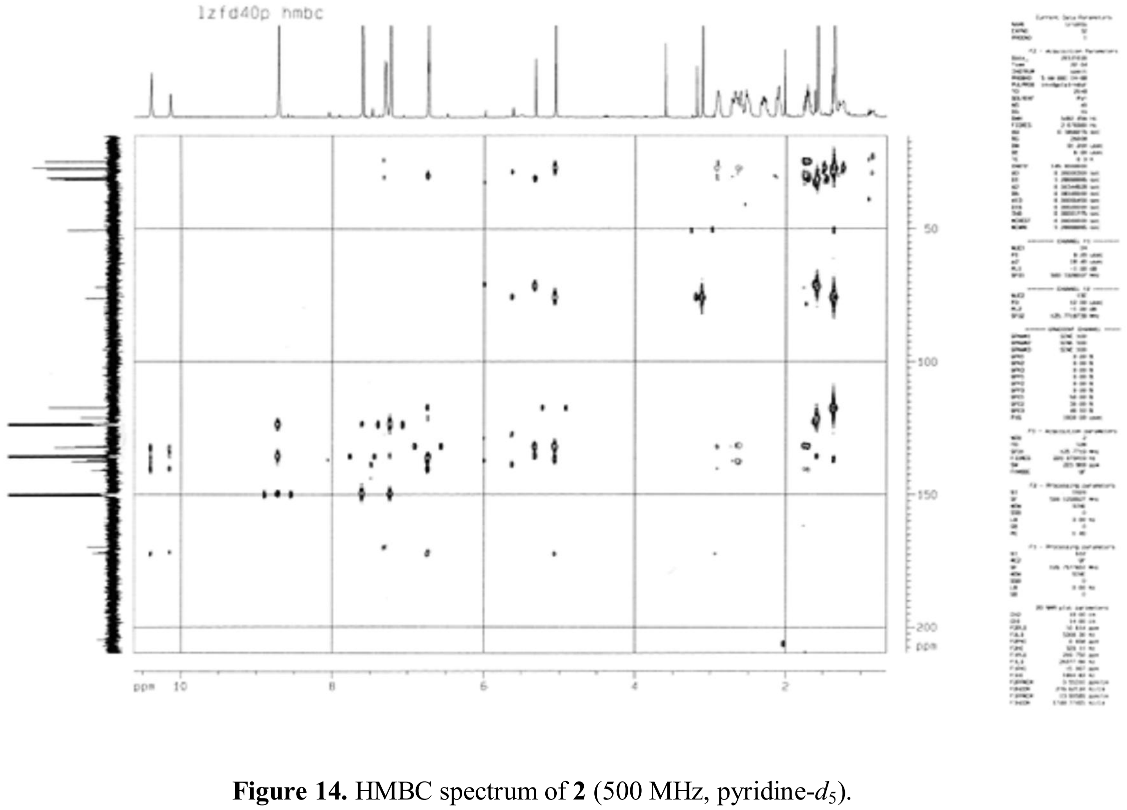

Figure 14. HMBC spectrum of **2** (500 MHz, pyridine-d_5).

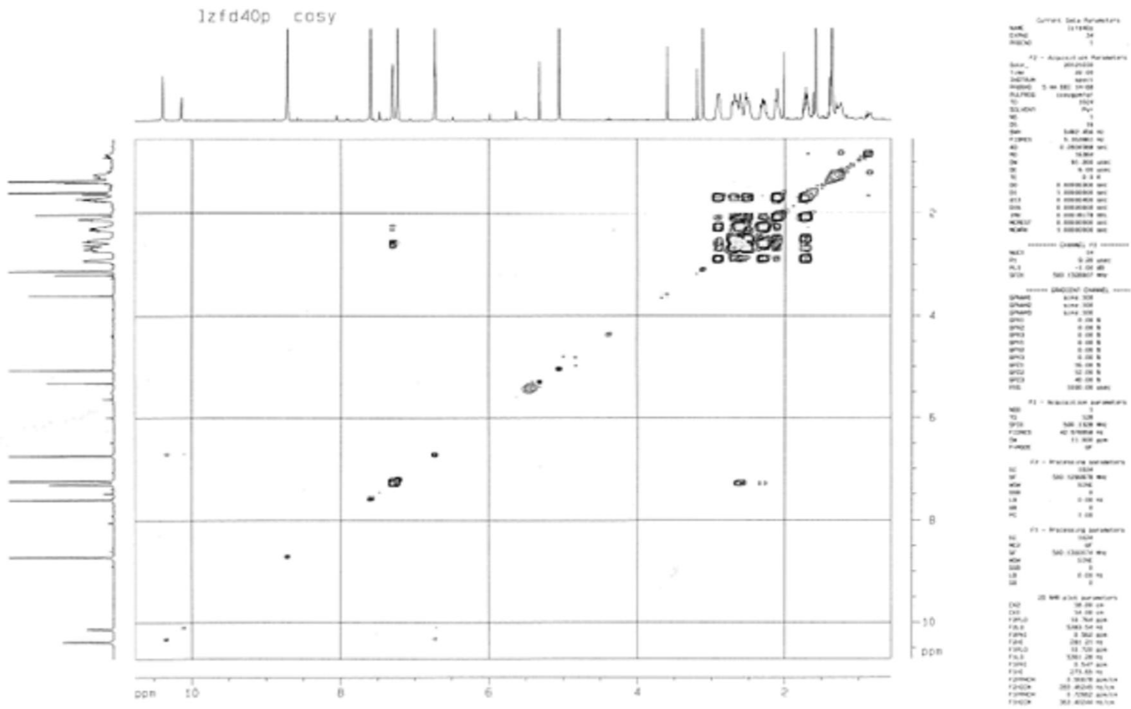

Figure 15. COSY spectrum of **2** (500 MHz, pyridine-d_5).

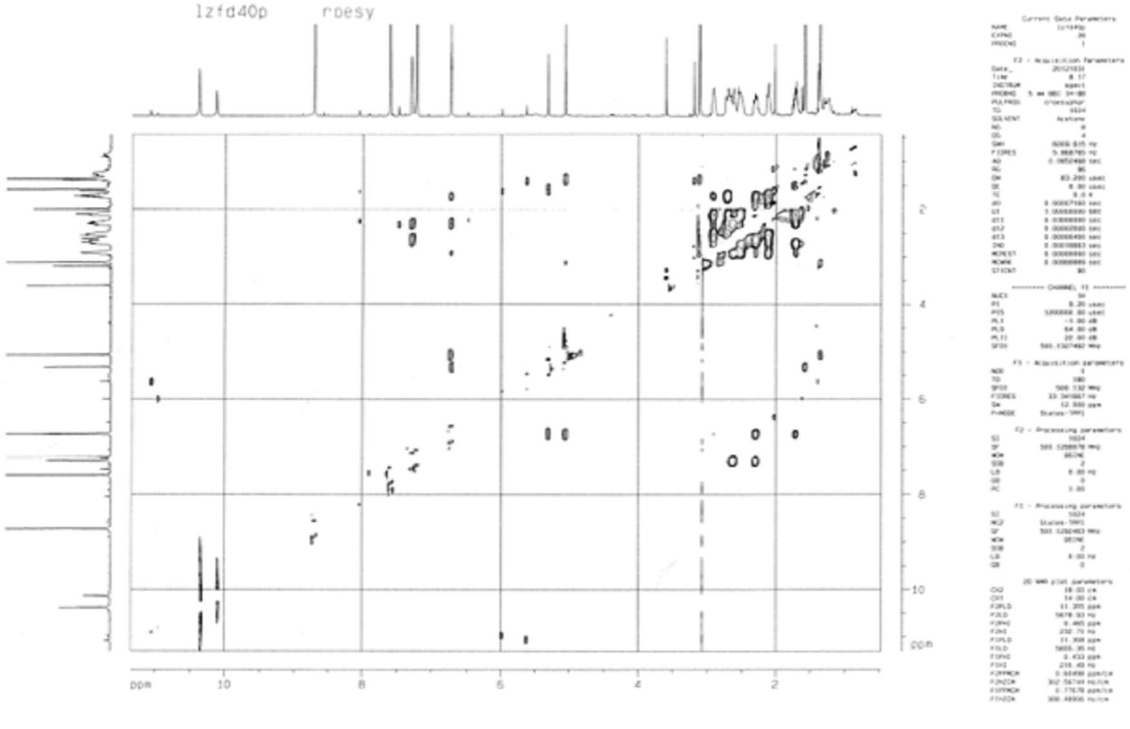

Figure 16. ROESY spectrum of **2** (500 MHz, pyridine-d_5).

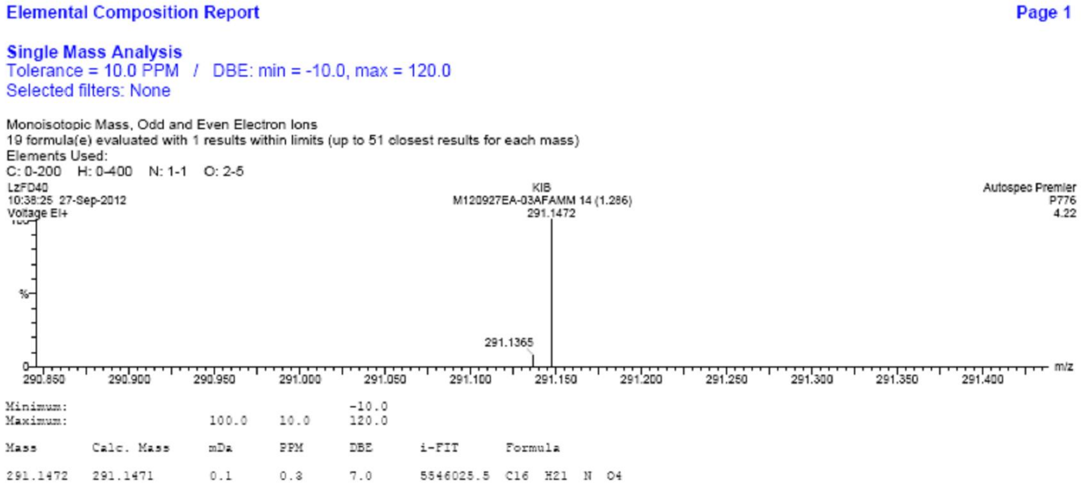

Figure 17. HR-EI-MS of **2**.

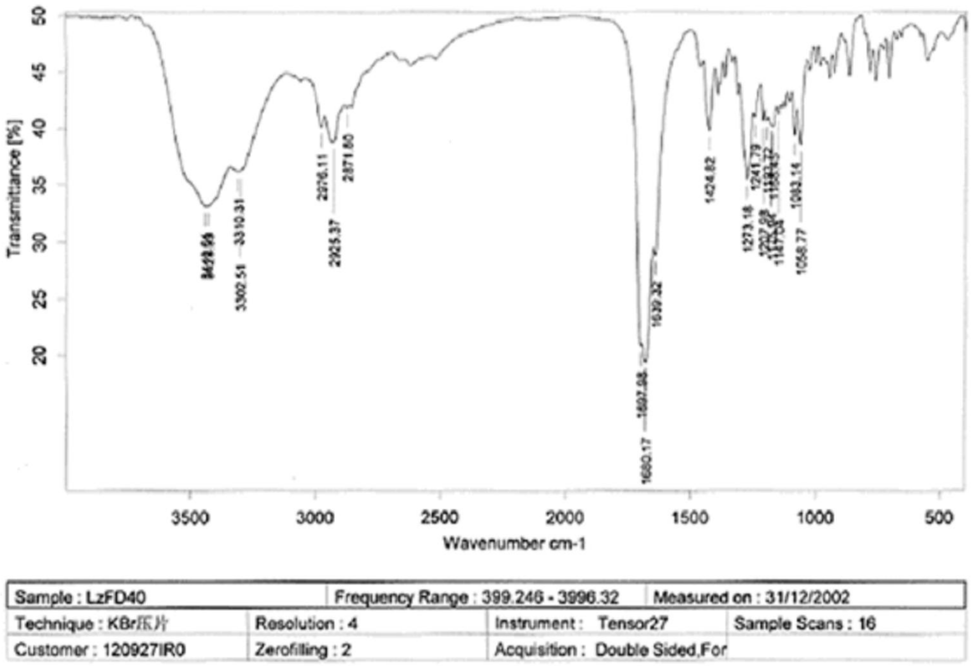

Figure 18. IR spectrum of **2**.

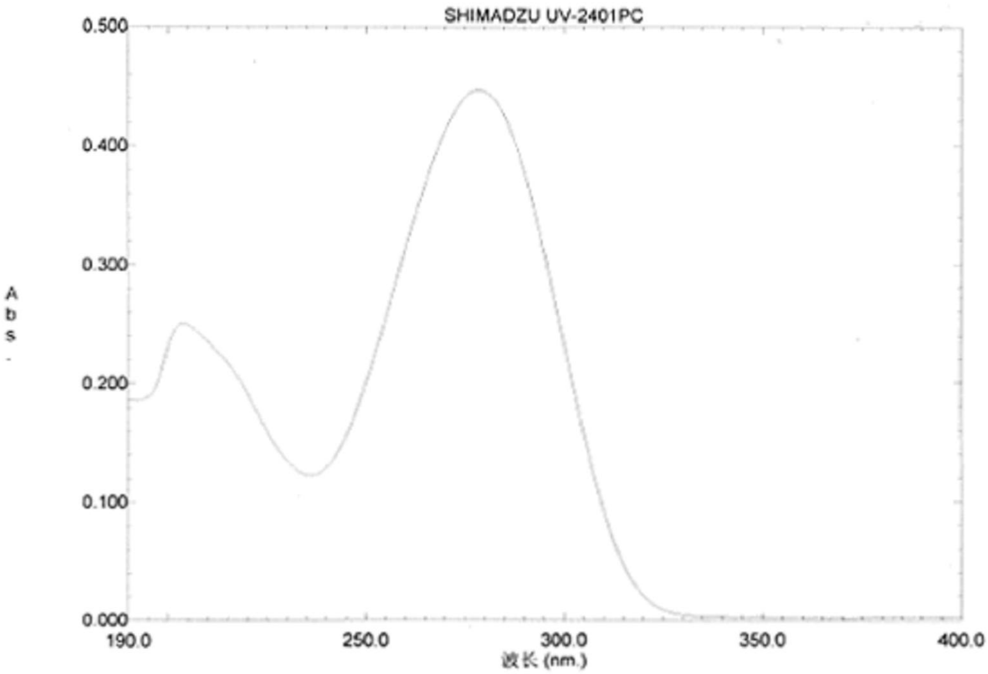

文件名: LZFD40

LZFD40

样品浓度: 0.0059毫克/毫升
溶剂: 甲醇

创建于: 17:33 12-10-15
数据: 原始

测量模式: Abs.
扫描速度: 中速
狭缝: 5.0
采样间隔: 0.2

峰.	波长 (nm.)	Abs.
1	278.60	0.4462
2	203.60	0.2500

Figure 19. UV spectrum of **2**.

Optical rotation measurement

Model : P-1020 (A060460638)

No.	Sample	Mode	Data	Monitor<br>Blank	Temp.<br>Cell<br>Temp Point	Date<br>Comment<br>Sample Name	Light<br>Filter<br>Operator	Cycle Time<br>Integ Time
No.1	5 (1/3)	Sp.Rot	-111.2310	-0.0723<br>0.0000	21.8<br>50.00<br>Cell	Mon Oct 15 14:27:48 2012<br>0.00130g/mlMeOH<br>LZFD40	Na<br>589nm	2 sec<br>10 sec
No.2	5 (2/3)	Sp.Rot	-111.3850	-0.0724<br>0.0000	21.8<br>50.00<br>Cell	Mon Oct 15 14:28:01 2012<br>0.00130g/mlMeOH<br>LZFD40	Na<br>589nm	2 sec<br>10 sec
No.3	5 (3/3)	Sp.Rot	-114.9230	-0.0747<br>0.0000	21.8<br>50.00<br>Cell	Mon Oct 15 14:28:14 2012<br>0.00130g/mlMeOH<br>LZFD40	Na<br>589nm	2 sec<br>10 sec

-112.5128°

Figure 20. Optical rotation measurement of **2**.

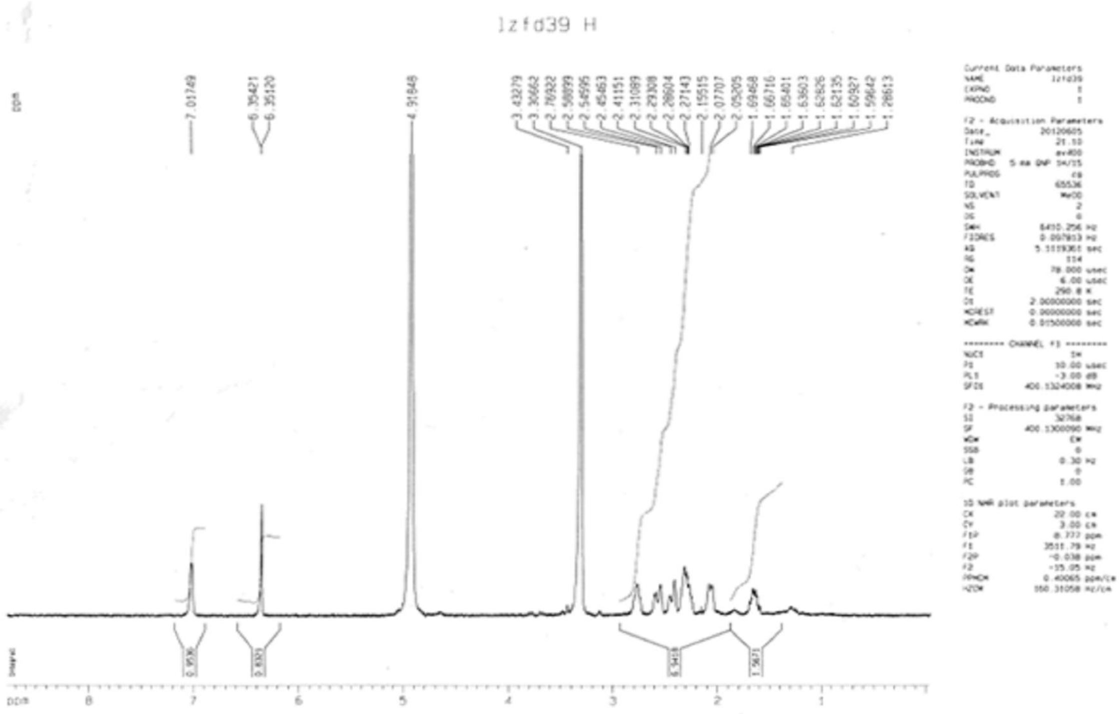

Figure 21. ^{1}H NMR spectrum of **3** (400 MHz, methanol-d_4).

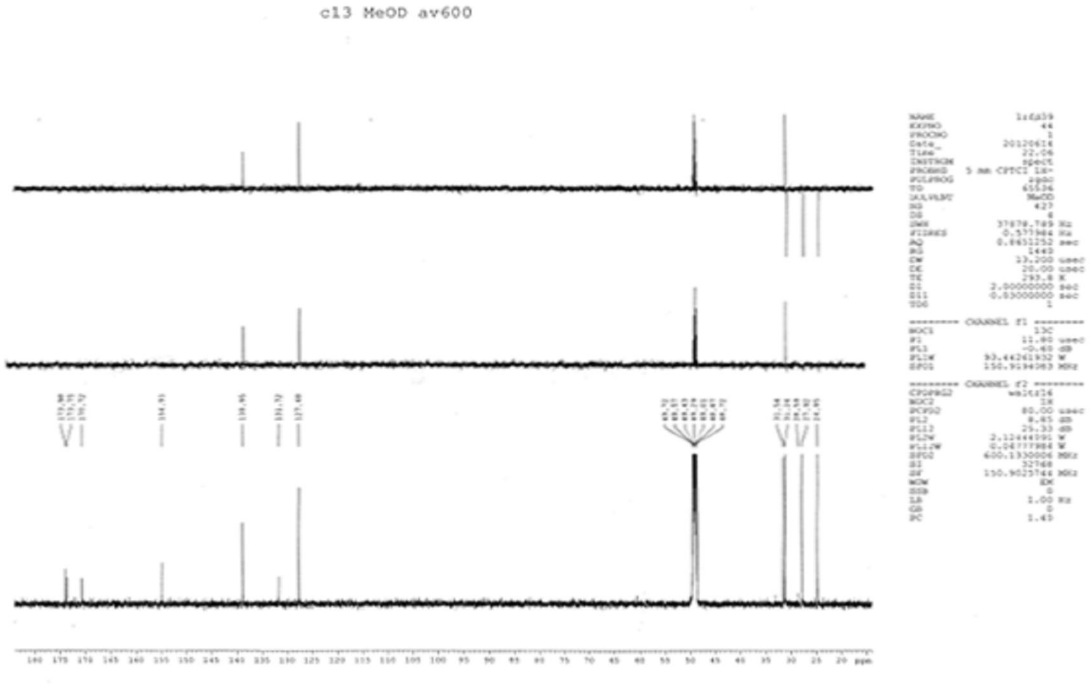

Figure 22. ^{13}C NMR spectrum of **3** (150 MHz, methanol-d_4).

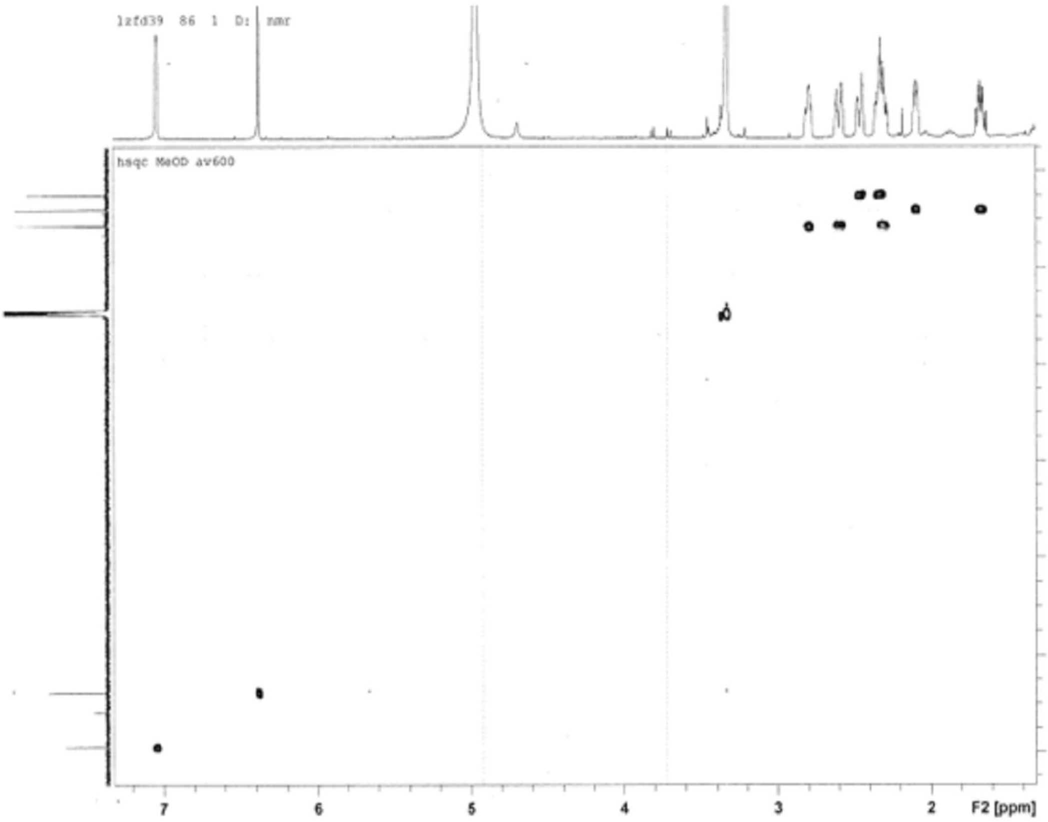

Figure 23. HSQC spectrum of **3** (600 MHz, methanol-d_4).

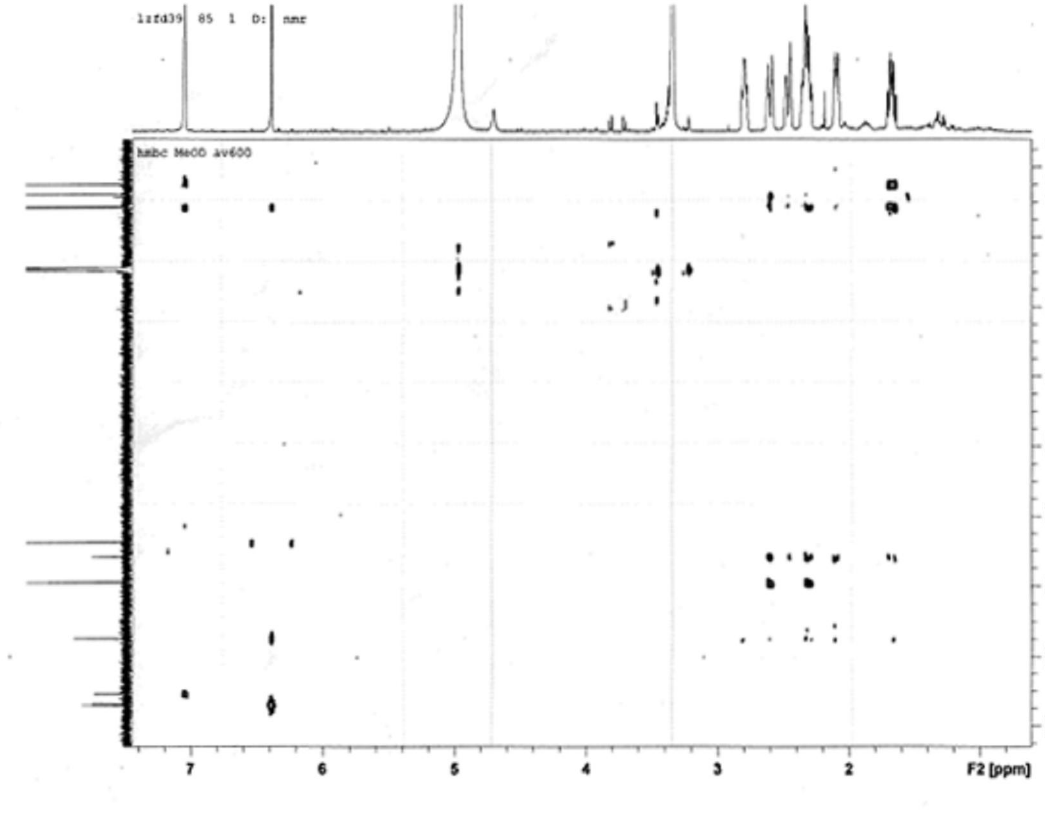

Figure 24. HMBC spectrum of **3** (600 MHz, methanol-d_4).

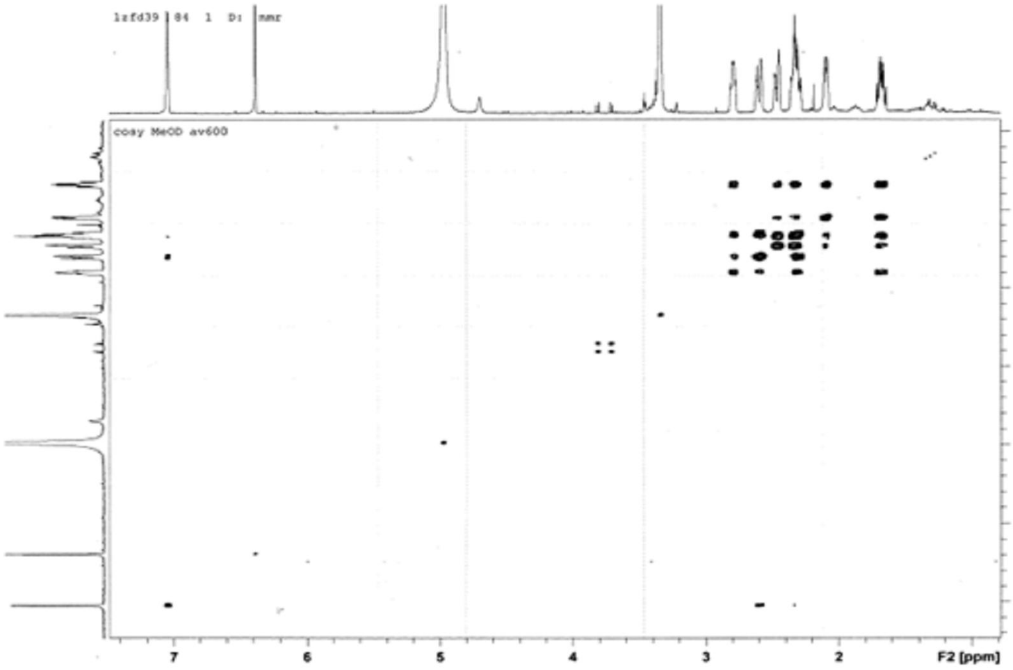

Figure 25. COSY spectrum of **3** (600 MHz, methanol-d_4).

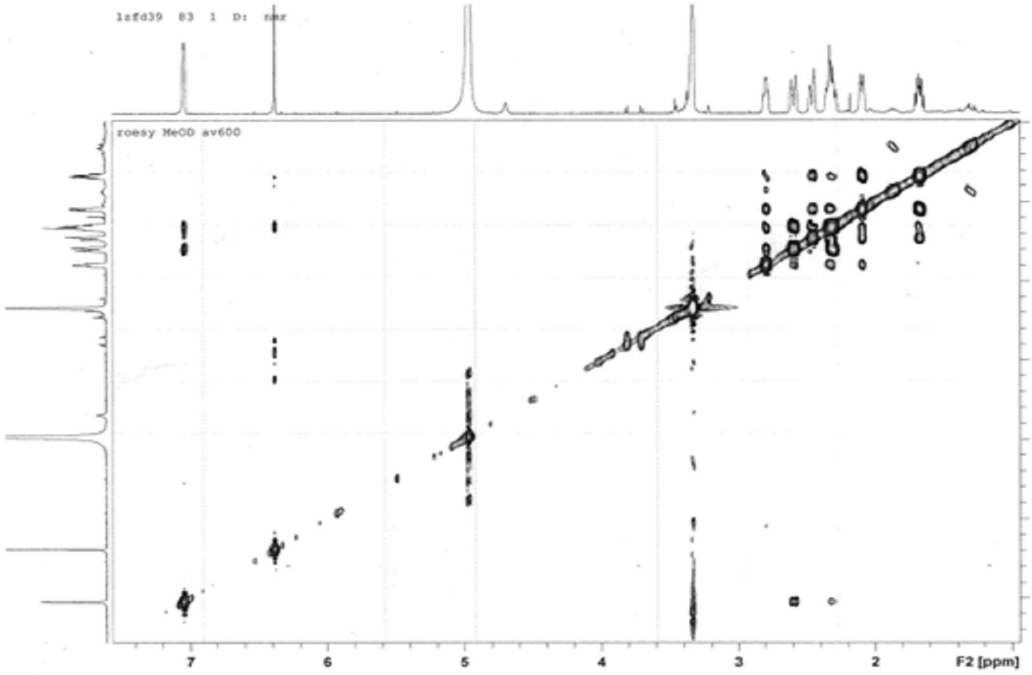

Figure 26. ROESY spectrum of **3** (600 MHz, methanol-d_4).

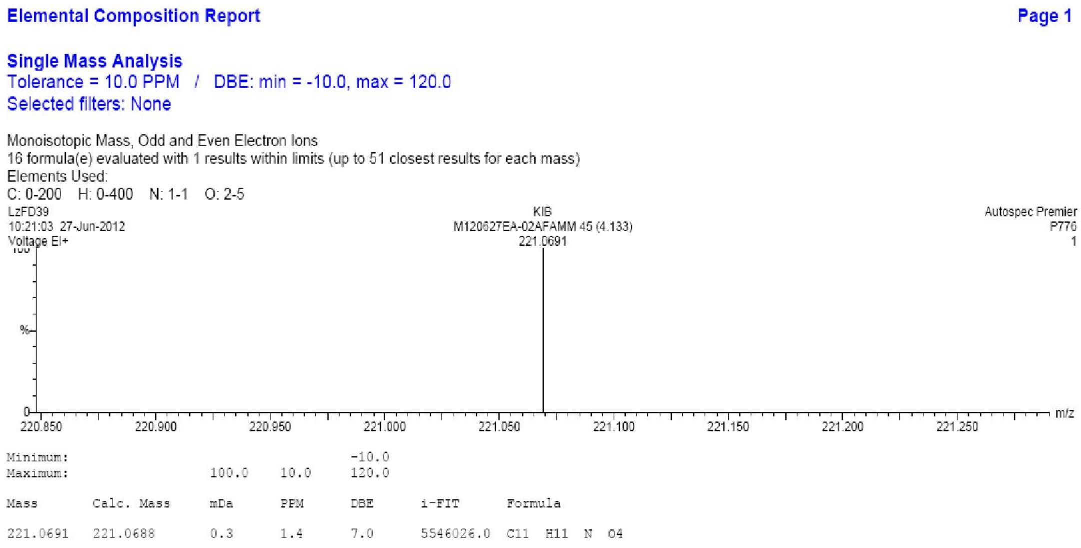

Figure 27. HR-EI-MS of **3**.

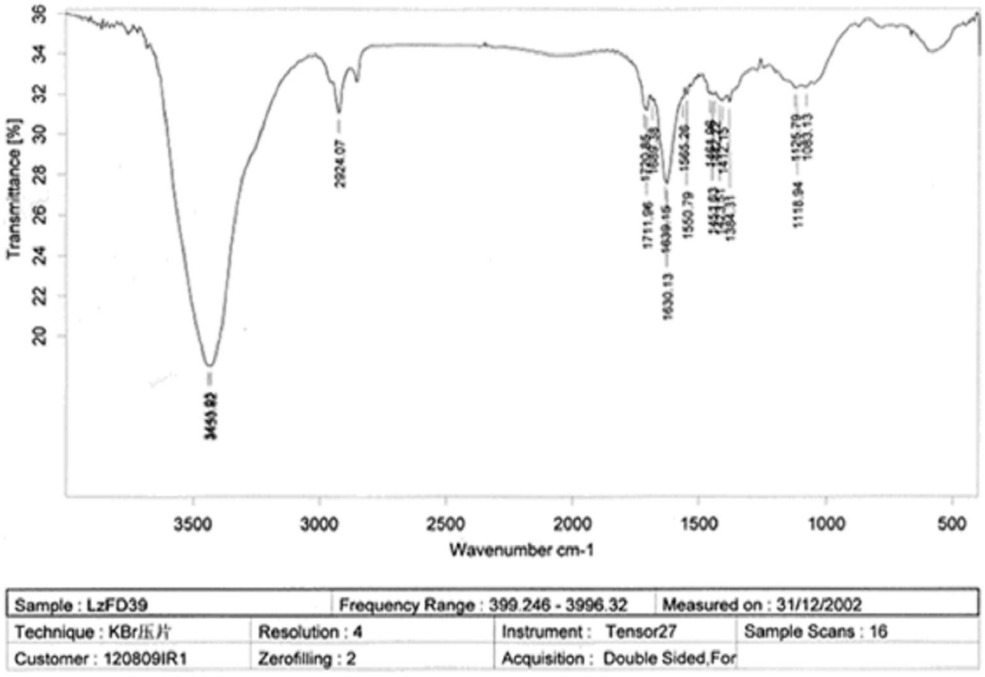

Figure 28. IR spectrum of **3**.

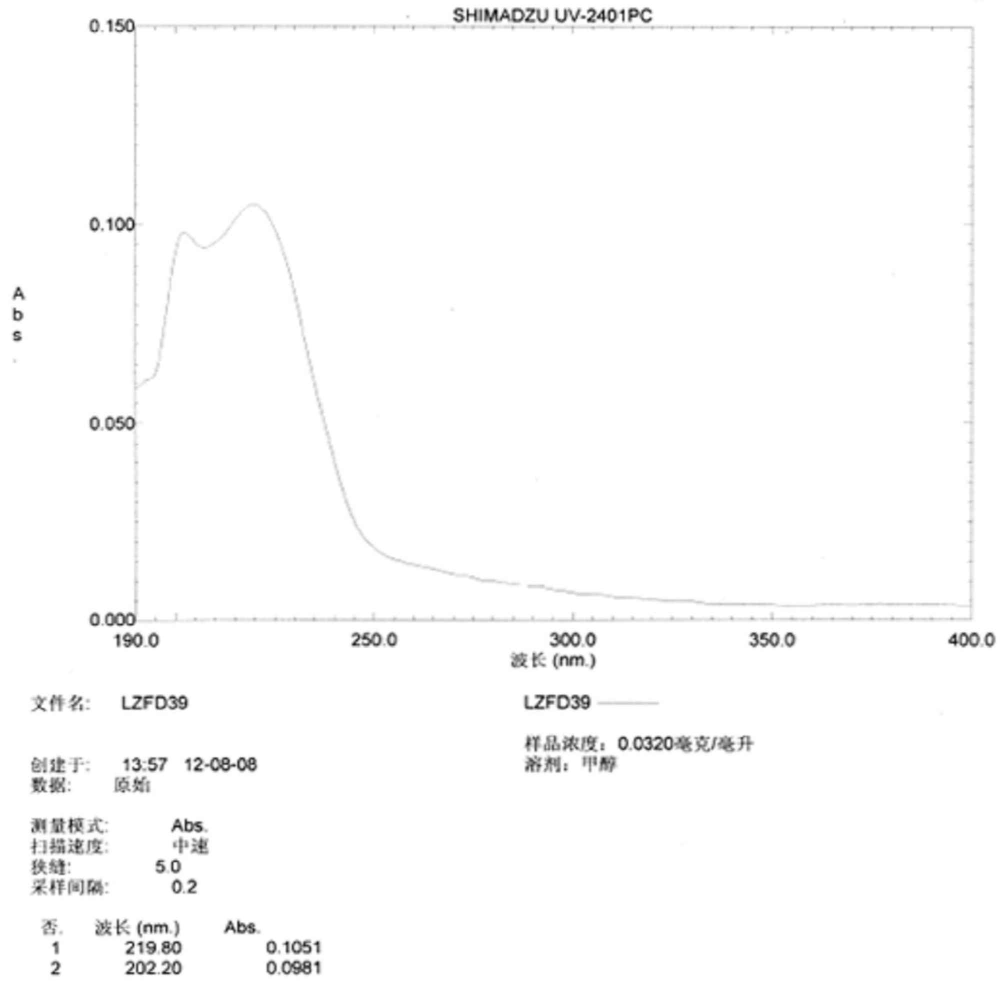

Figure 29. UV spectrum of **3**.

Optical rotation measurement

Model : P-1020 (A060460638)

No.	Sample	Mode	Data	Monitor<br>Blank	Temp.<br>Cell<br>Temp Point	Date<br>Comment<br>Sample Name	Light<br>Filter<br>Operator	Cycle Time<br>Integ Time	
No.1	1 (1/3)	Sp.Rot	-27.2500	-0.0109<br>0.0000	24.6<br>50.00<br>Cell	Wed Aug 08 13:22:11 2012<br>0.00080g/mlMeOH<br>LZFD39	Na<br>589nm	2 sec<br>10 sec	
No.2	1 (2/3)	Sp.Rot	-23.7500	-0.0095<br>0.0000	24.6<br>50.00<br>Cell	Wed Aug 08 13:22:25 2012<br>0.00080g/mlMeOH<br>LZFD39	Na<br>589nm	2 sec<br>10 sec	-24.7500°
No.3	1 (3/3)	Sp.Rot	-23.2500	-0.0093<br>0.0000	24.5<br>50.00<br>Cell	Wed Aug 08 13:22:38 2012<br>0.00080g/mlMeOH<br>LZFD39	Na<br>589nm	2 sec<br>10 sec	

Figure 30. Optical rotation measurement of **3**.

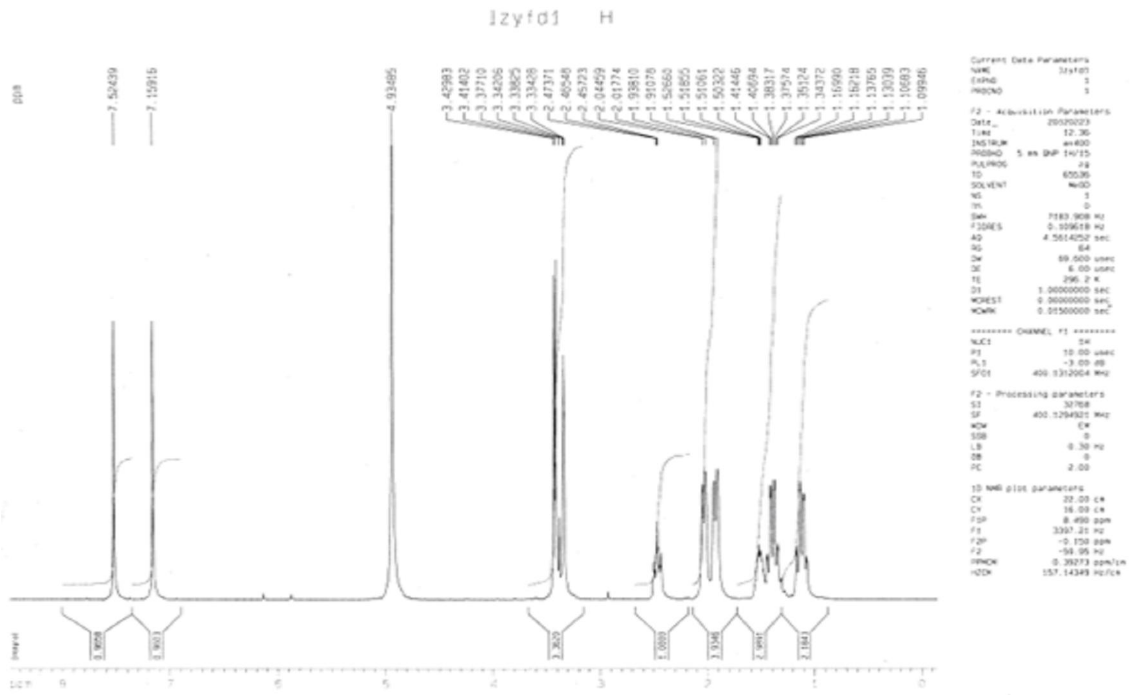

Figure 31. ^{1}H NMR spectrum of **4** (400 MHz, methanol-d_4).

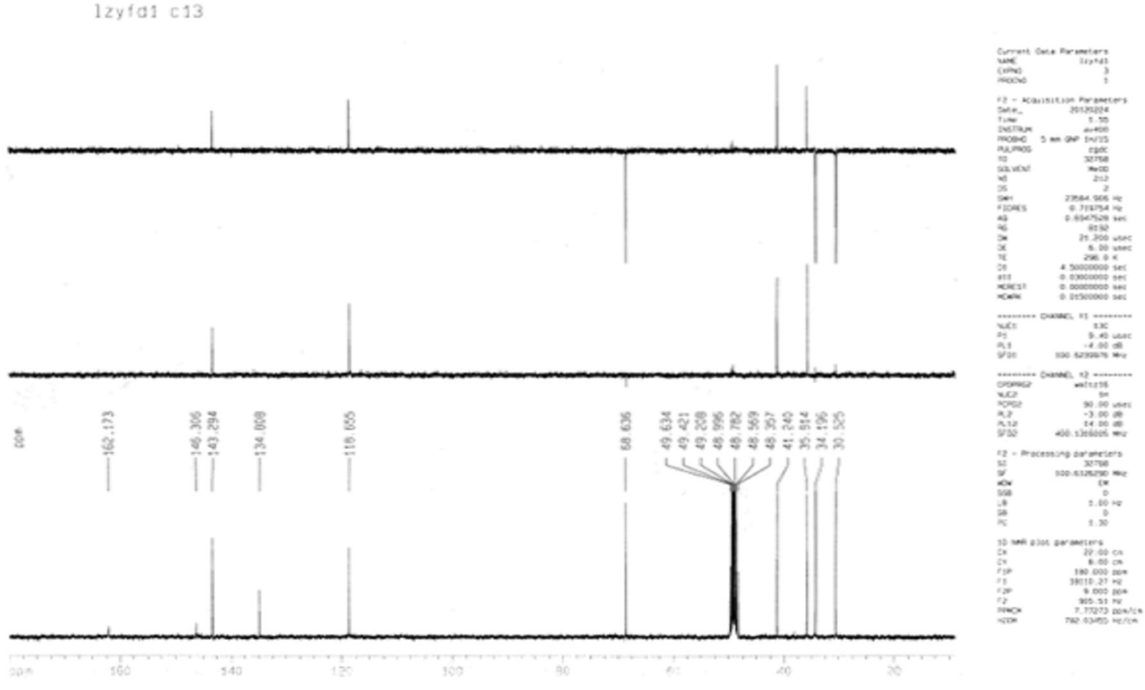

Figure 32. ^{13}C NMR spectrum of **4** (100 MHz, methanol-d_4).

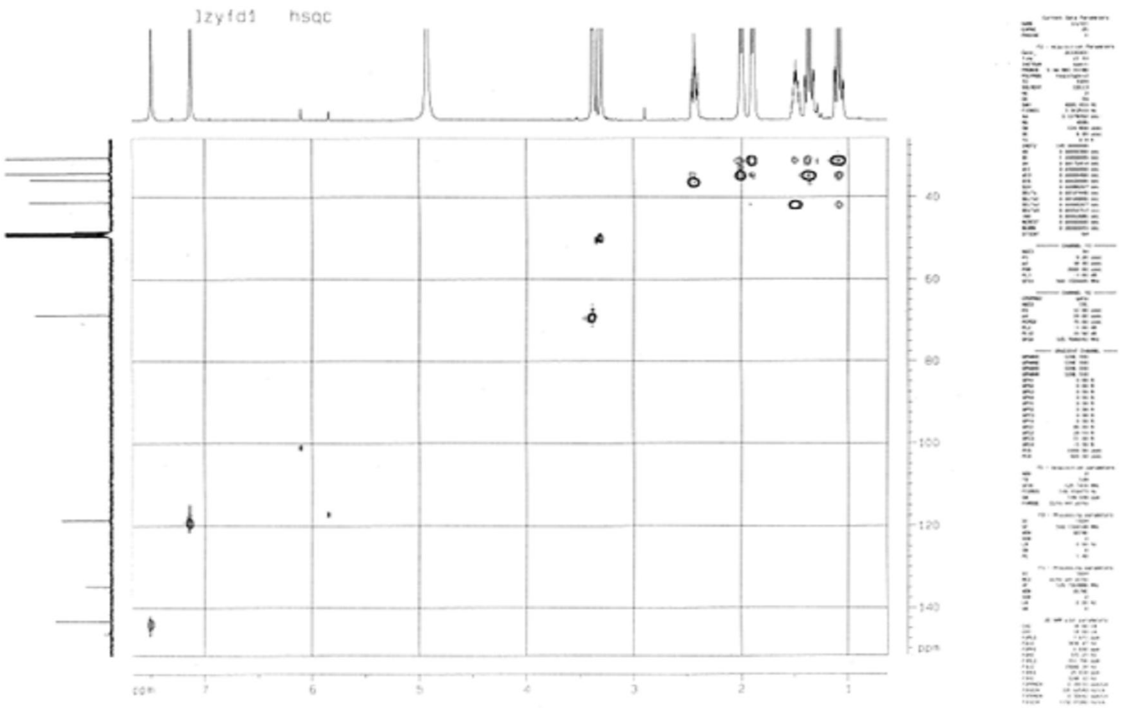

Figure 33. HSQC spectrum of **4** (500 MHz, methanol-d_4).

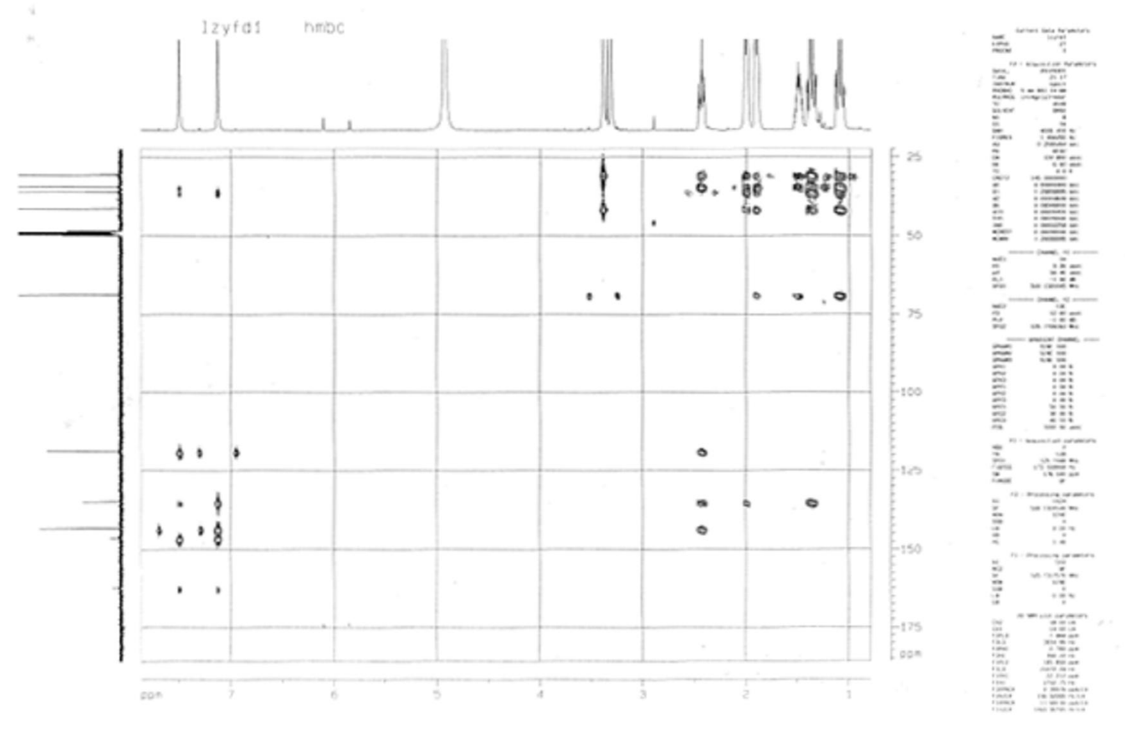

Figure 34. HMBC spectrum of **4** (500 MHz, methanol-d_4).

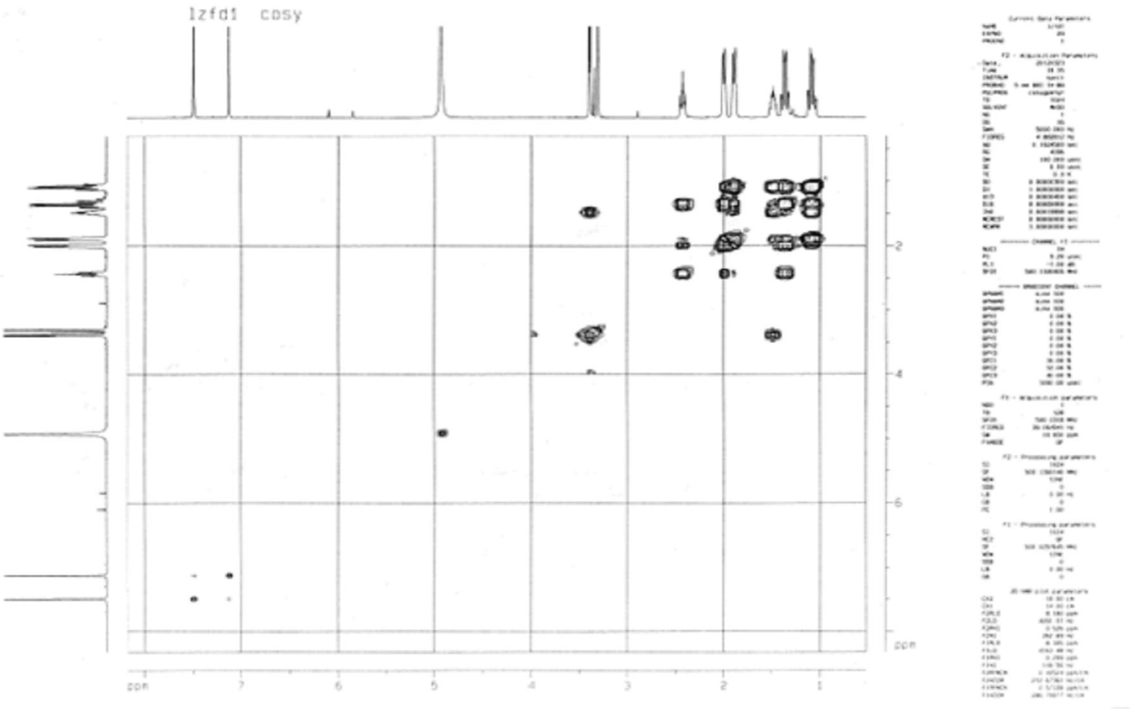

Figure 35. COSY spectrum of **4** (500 MHz, methanol-d_4).

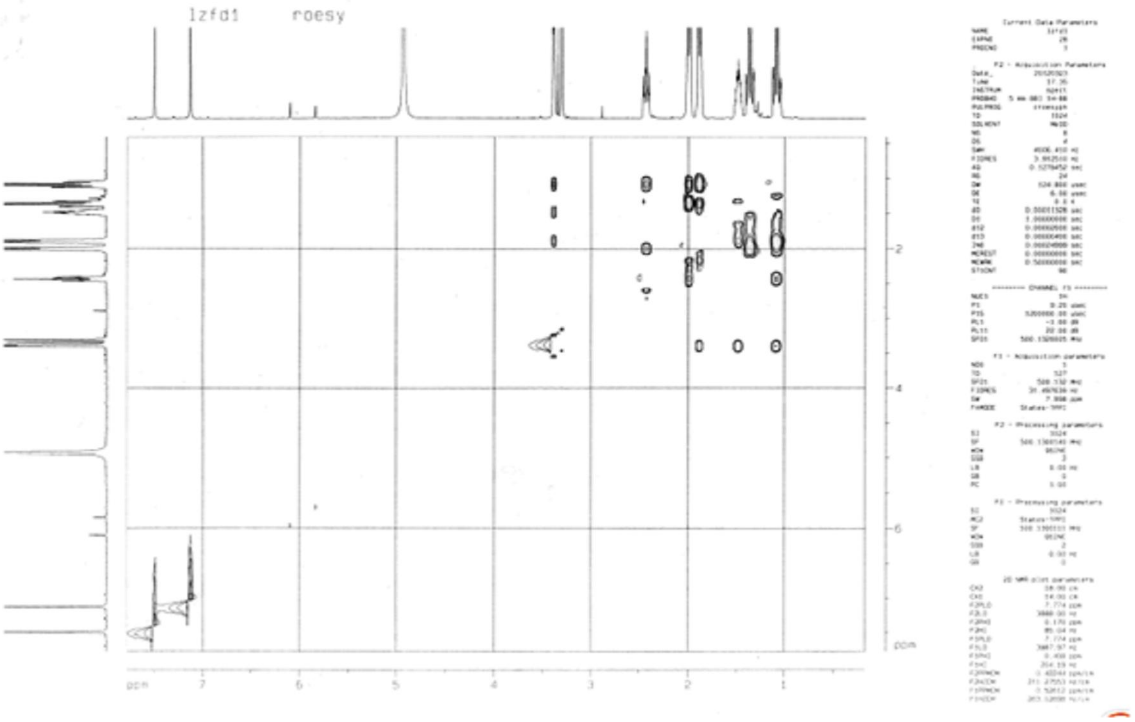

Figure 36. ROESY spectrum of **4** (500 MHz, methanol-d_4).

Elemental composition calculator

```
Target m/z:        +225.1127      amu
Tolerance:  +10.0000      ppm
Result type:      Elemental
Max num of results:       1000
Min DBE:    -10.0000      Max DBE:          +60.0000
Electron state:   OddAndEven
Num of charges:   0
Add water:N/A
Add proton:       N/A
File Name:        120306ESIA    LzyFD1.wiff
```

	Elements	Min Number	Max Number:
1	2H	0	0
2	Br	0	0
3	C	0	200
4	Cl	0	0
5	F	0	0
6	H	0	400
7	I	0	0
8	K	0	0
9	N	0	0
10	Na	0	0
11	O	3	6
12	P	0	0
13	Pt	0	0
14	S	0	0
15	Si	0	0

	Formula	Calculated m/z (amu)	mDa Error	PPM Error	DBE
1	C12 H17 O4	225.1126	0.0157	0.0700	4.5

Figure 37. HR-ESI-MS of **4**.

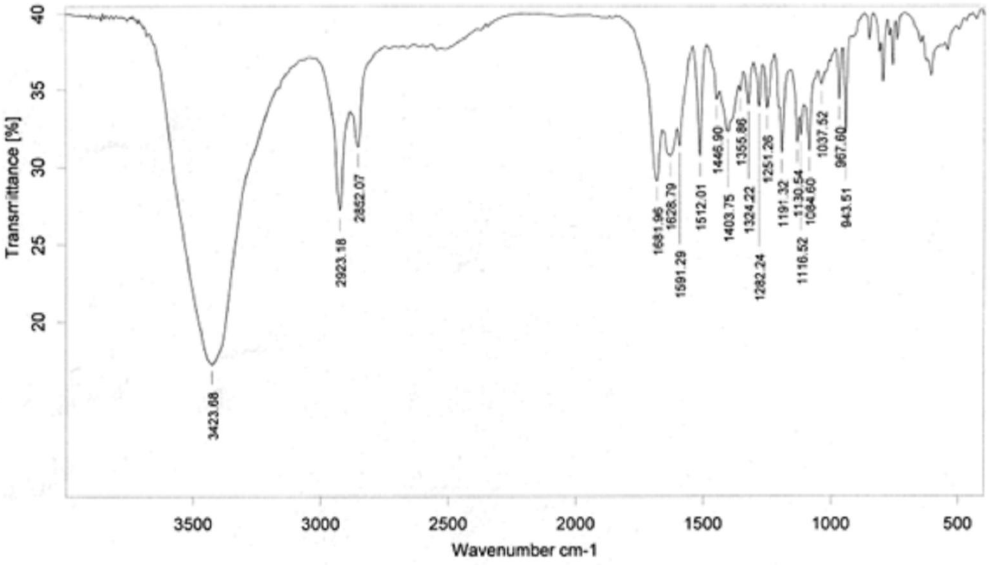

Sample : LzyFD1	Frequency Range : 399.246 - 3996.32	Measured on : 28/03/2012	
Technique : KBr压片	Resolution : 4	Instrument : Tensor27	Sample Scans : 16
Customer : 120328IR5	Zerofilling : 2	Acquisition : Double Sided,For	

Figure 38. IR spectrum of **4**.

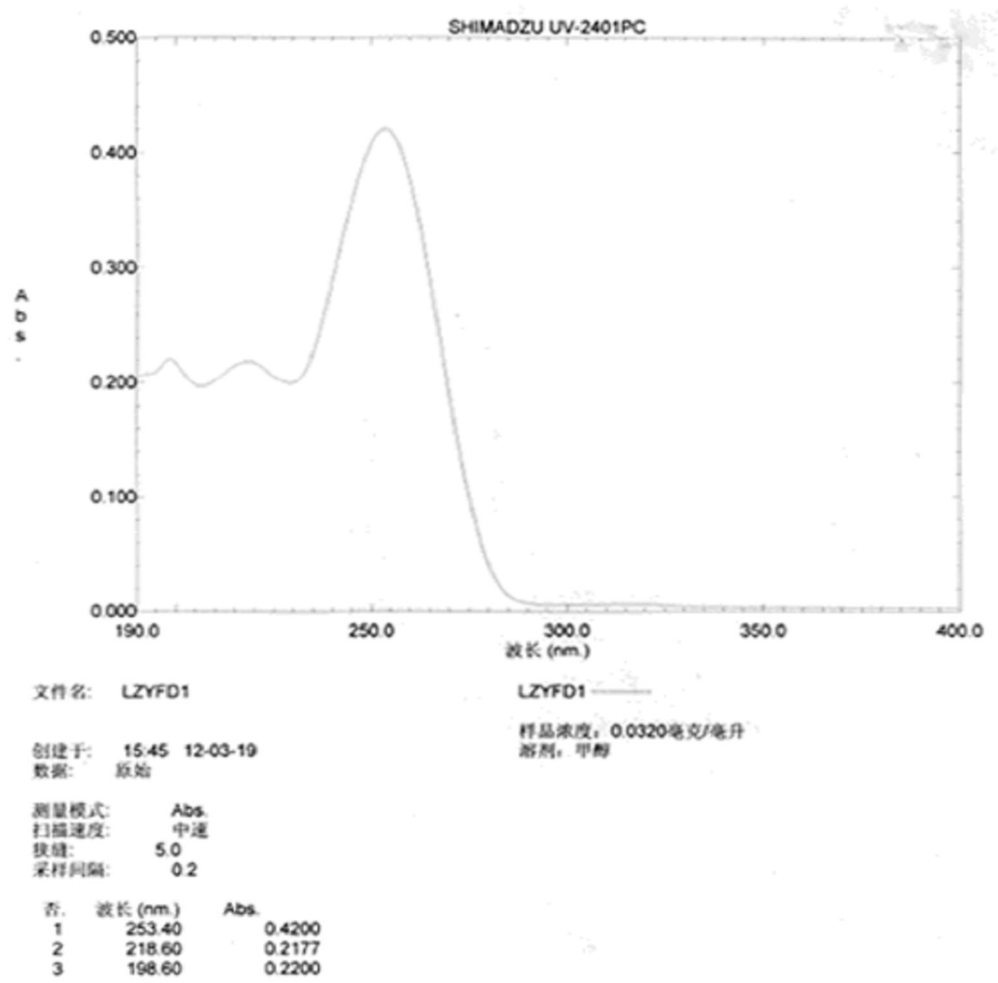

Figure 39. UV spectrum of **4**.

Optical rotation measurement

Model : P-1020 (A060460638)

No.	Sample	Mode	Data	Monitor<br>Blank	Temp.<br>Cell<br>Temp Point	Date<br>Comment<br>Sample Name	Light<br>Filter<br>Operator	Cycle Time<br>Integ Time
No.1	3 (1/3)	Sp.Rot	-10.6000	-0.0053<br>0.0000	19.7<br>50.00<br>Cell	Mon Mar 19 15:14:22 2012<br>0.00100g/mlMeOH<br>LZYFD1	Na<br>589nm	2 sec<br>10 sec
No.2	3 (2/3)	Sp.Rot	-12.8000	-0.0064<br>0.0000	19.7<br>50.00<br>Cell	Mon Mar 19 15:14:35 2012<br>0.00100g/mlMeOH<br>LZYFD1	Na<br>589nm	2 sec<br>10 sec
No.3	3 (3/3)	Sp.Rot	-10.0000	-0.0050<br>0.0000	19.7<br>50.00<br>Cell	Mon Mar 19 15:14:48 2012<br>0.00100g/mlMeOH<br>LZYFD1	Na<br>589nm	2 sec<br>10 sec

-11.1333°

Figure 40. Optical rotation measurement of **4**.

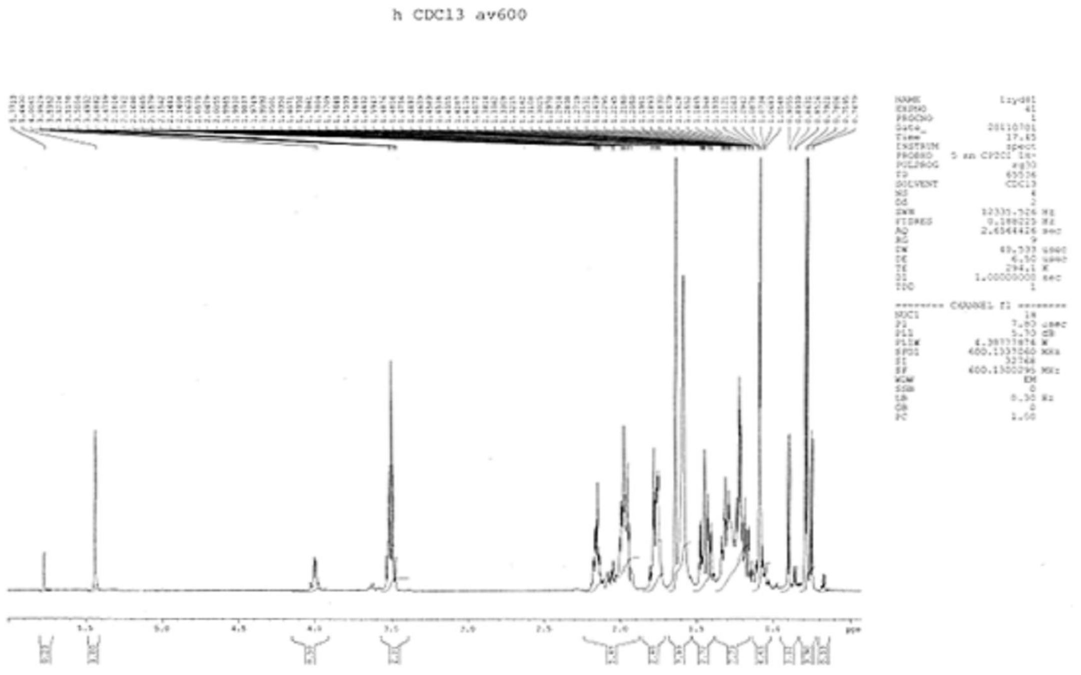

Figure 41. ^{1}H NMR spectrum of **5** (600 MHz, $CDCl_3$).

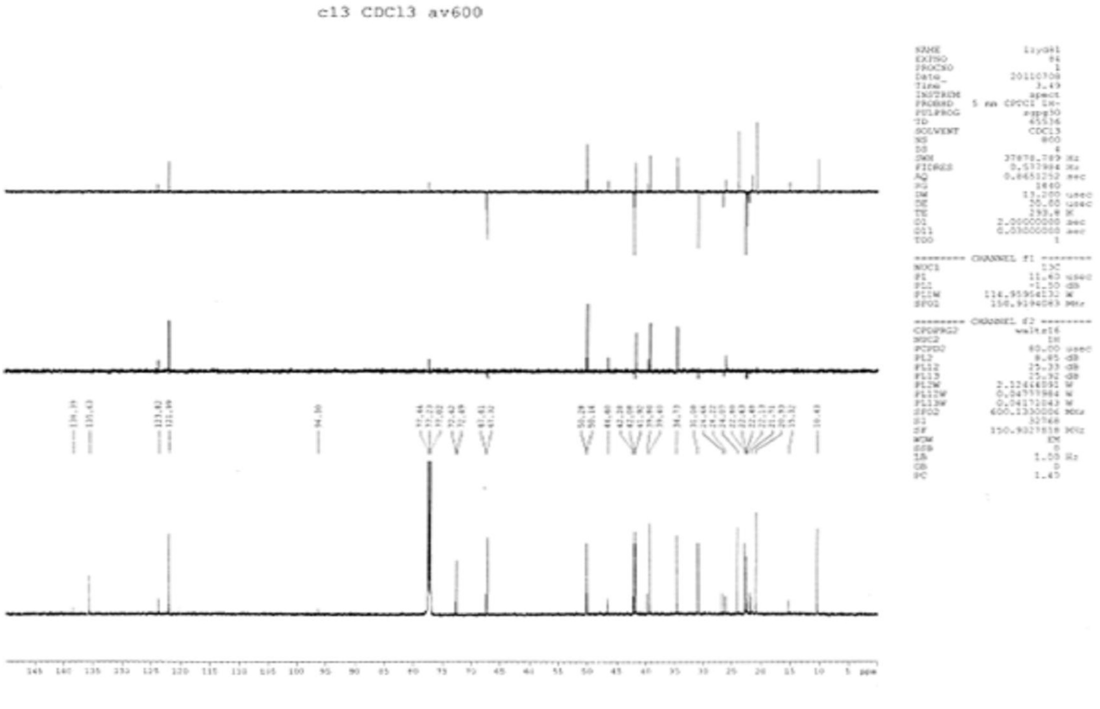

Figure 42. ^{13}C NMR spectrum of **5** (150 MHz, $CDCl_3$).

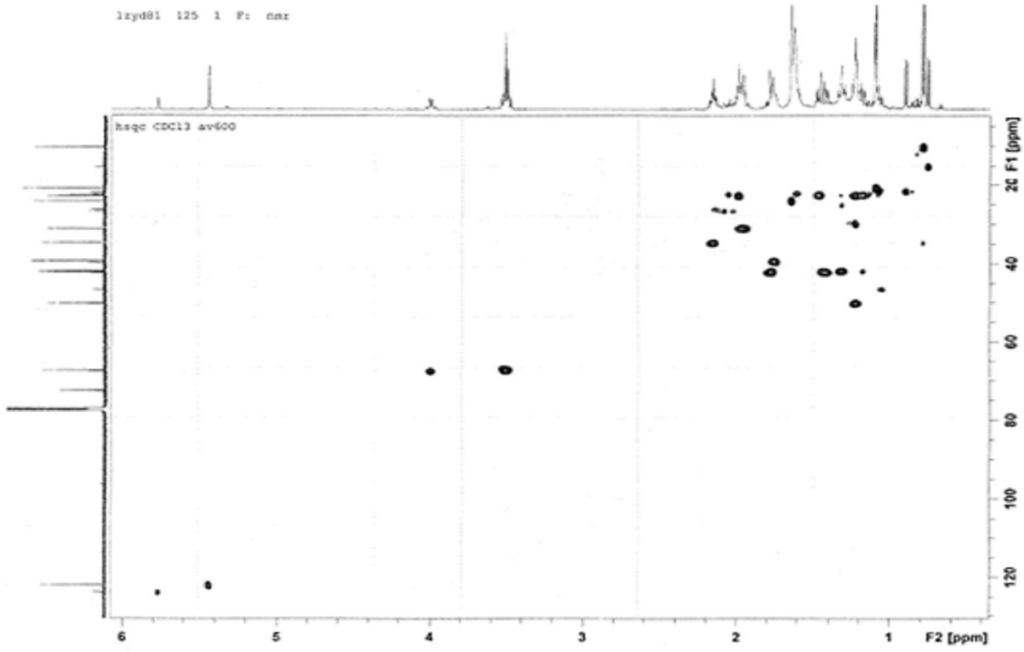

Figure 43. HSQC spectrum of **5** (600 MHz, $CDCl_3$).

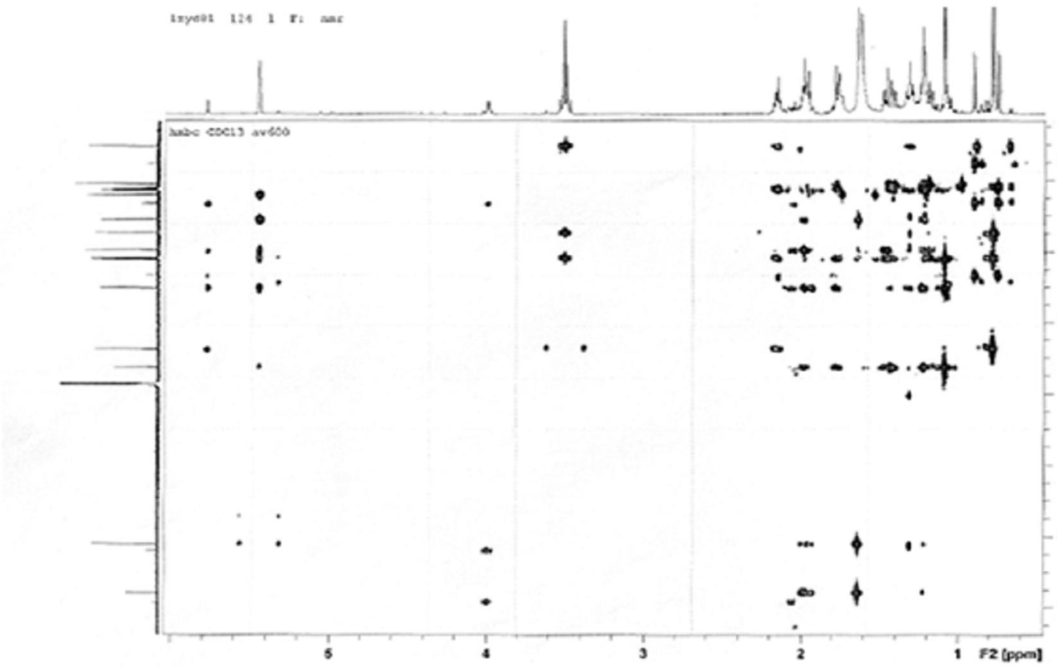

Figure 44. HMBC spectrum of **5** (600 MHz, $CDCl_3$).

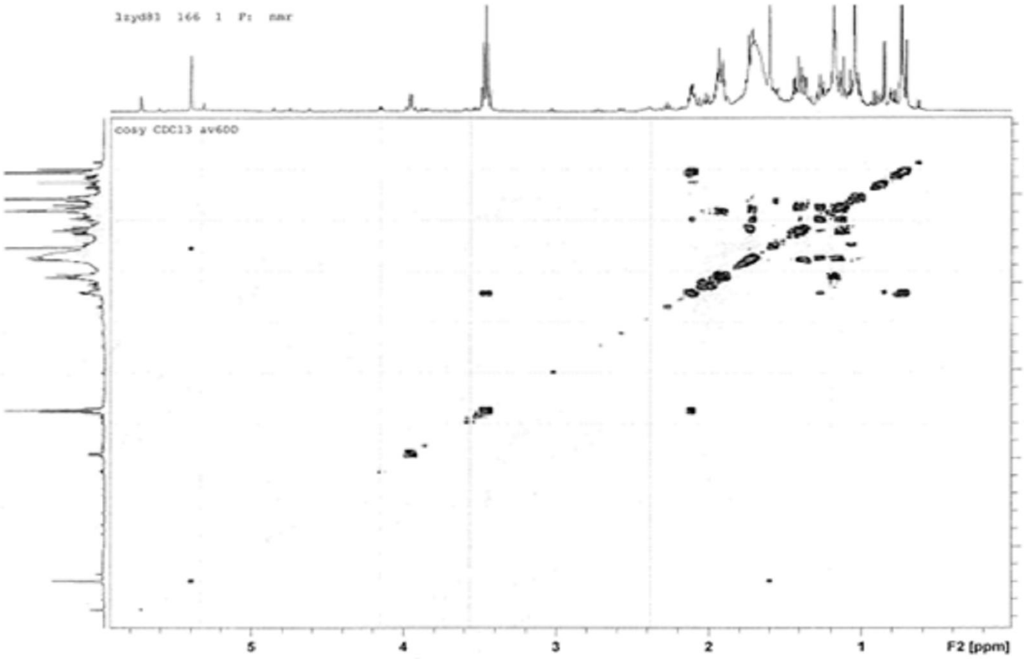

Figure 45. COSY spectrum of **5** (600 MHz, $CDCl_3$).

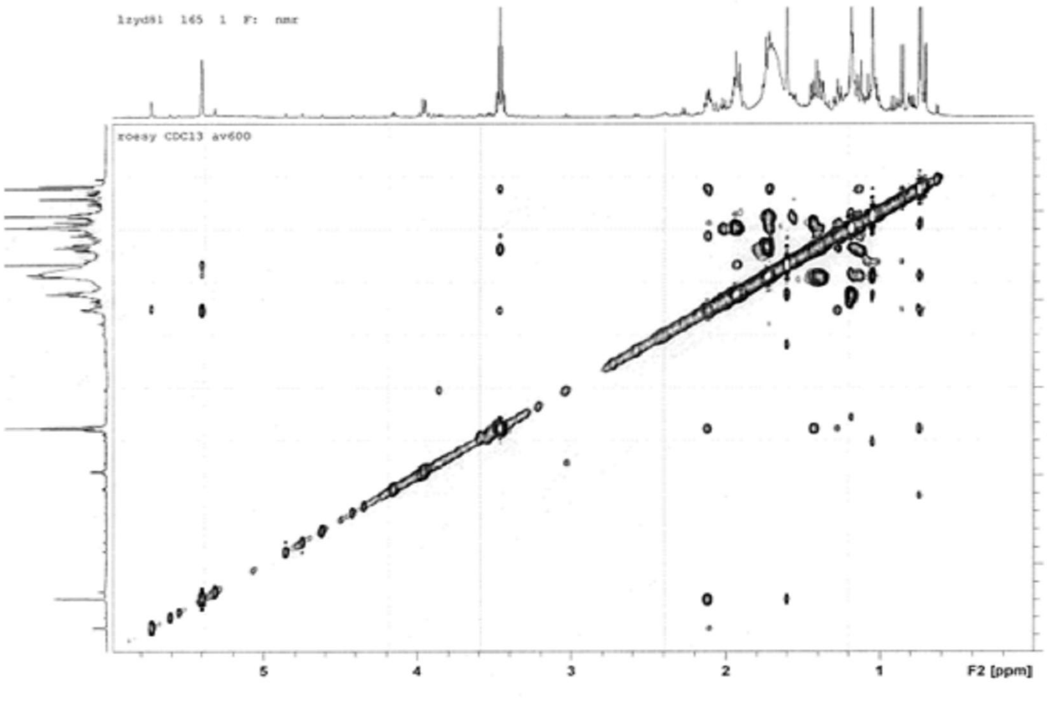

Figure 46. ROESY spectrum of **5** (600 MHz, $CDCl_3$).

Elemental composition calculator

Target m/z: +261.1827 amu
Tolerance: +10.0000 ppm
Result type: Elemental
Max num of results: 1000
Min DBE: -10.0000 Max DBE: +60.0000
Electron state: OddAndEven
Num of charges: 0
Add water: N/A
Add proton: N/A
File Name: 111108ESIA Lzyd8-1.wiff

	Elements	Min Number	Max Number
1	Br	0	0
2	C	0	200
3	Cl	0	0
4	F	0	0
5	H	0	400
6	I	0	0
7	K	0	0
8	N	0	0
9	Na	1	1
10	O	1	3
11	P	0	0
12	Pt	0	0
13	S	0	0
14	Si	0	0

	Formula	Calculated m/z (amu)	mDa Error	PPM Error	DBE
1	C15 H26 O2 Na	261.1830	-0.3500	-1.3401	2.5

Figure 47. HR-ESI-MS of **5**.

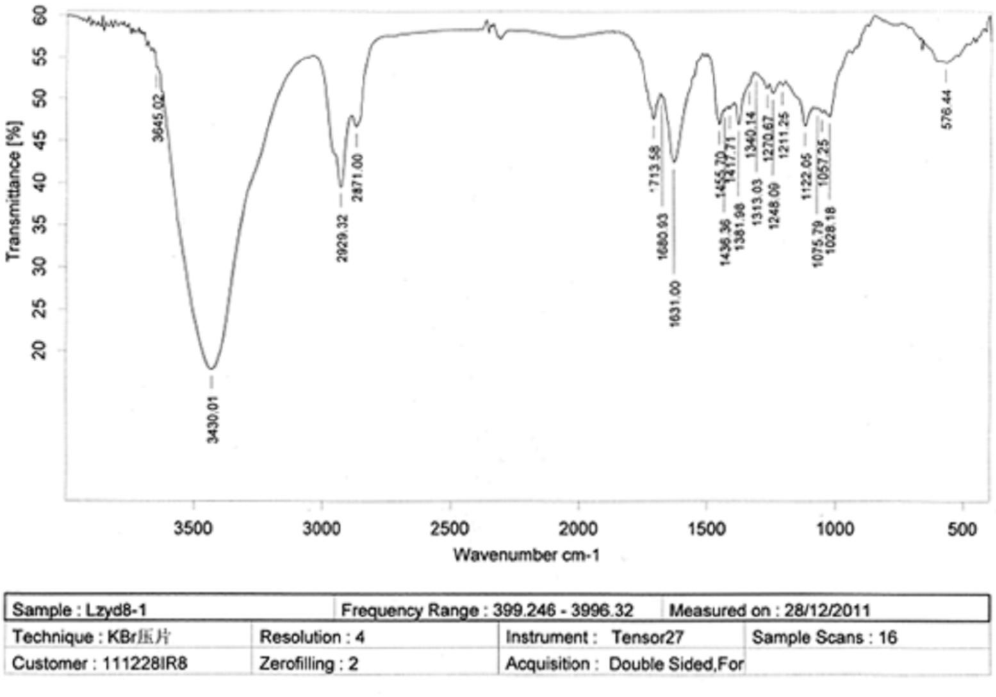

Figure 48. IR spectrum of **5**.

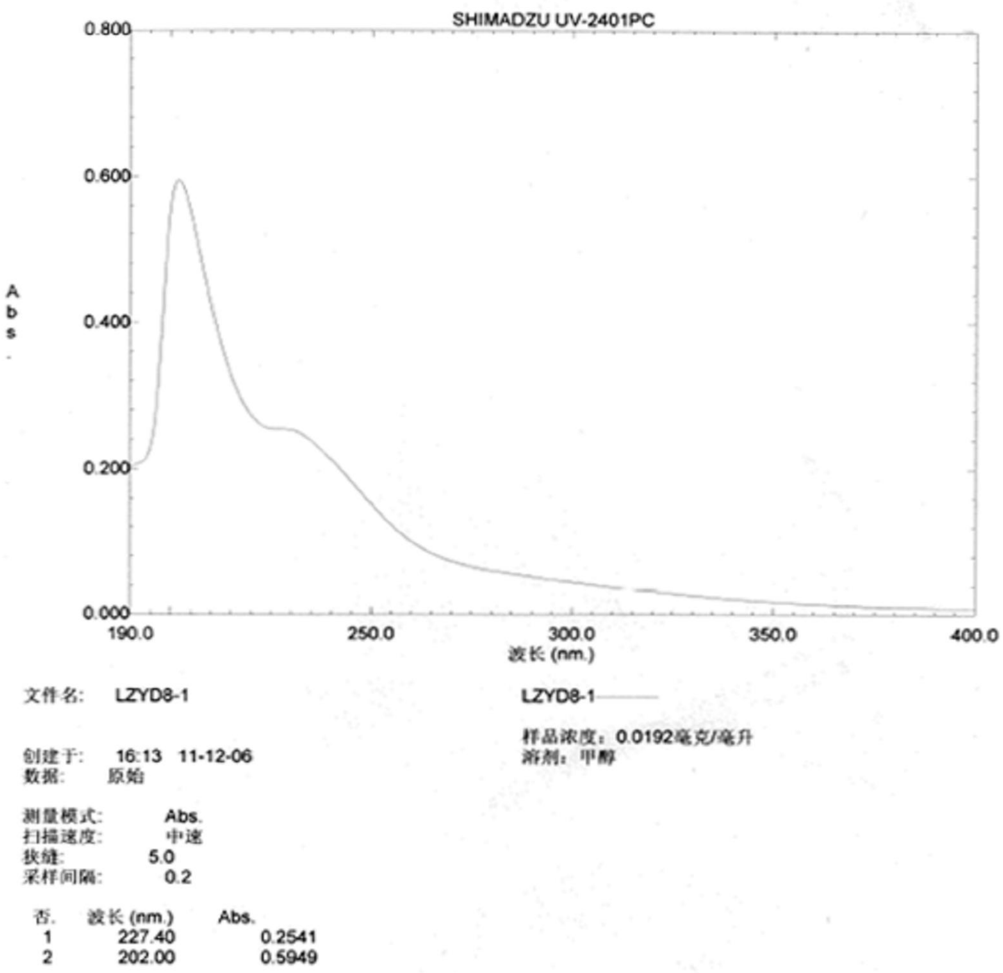

Figure 49. UV spectrum of **5**.

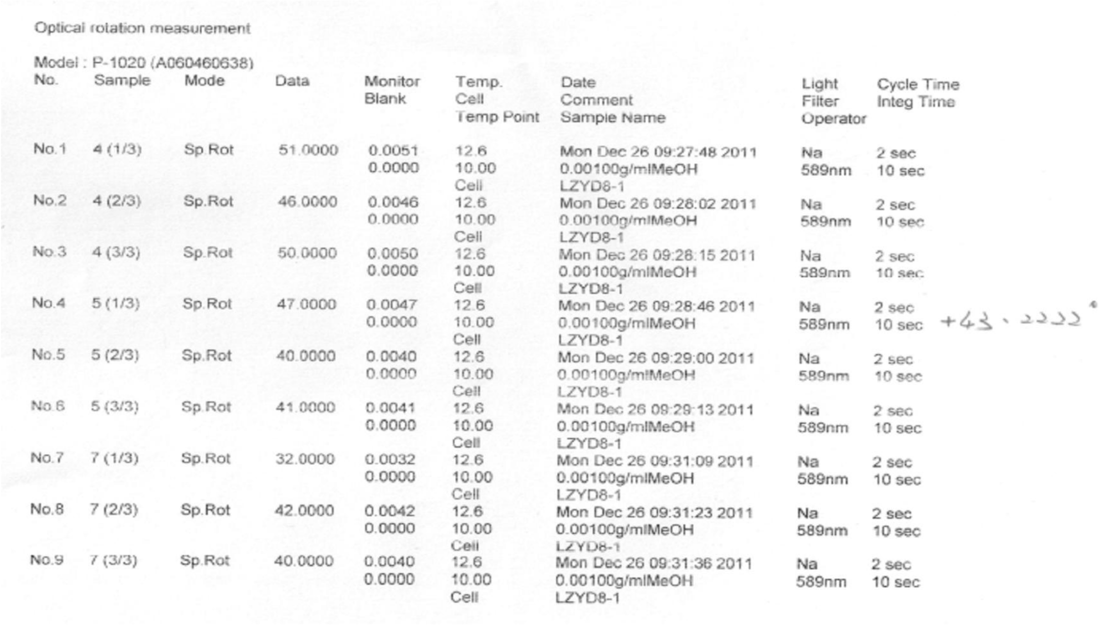
Optical rotation measurement

Model : P-1020 (A060460638)

No.	Sample	Mode	Data	Monitor<br>Blank	Temp.<br>Cell<br>Temp Point	Date<br>Comment<br>Sample Name	Light<br>Filter<br>Operator	Cycle Time<br>Integ Time
No.1	4 (1/3)	Sp.Rot	51.0000	0.0051<br>0.0000	12.6<br>10.00<br>Cell	Mon Dec 26 09:27:48 2011<br>0.00100g/mlMeOH<br>LZYD8-1	Na<br>589nm	2 sec<br>10 sec
No.2	4 (2/3)	Sp.Rot	46.0000	0.0046<br>0.0000	12.6<br>10.00<br>Cell	Mon Dec 26 09:28:02 2011<br>0.00100g/mlMeOH<br>LZYD8-1	Na<br>589nm	2 sec<br>10 sec
No.3	4 (3/3)	Sp.Rot	50.0000	0.0050<br>0.0000	12.6<br>10.00<br>Cell	Mon Dec 26 09:28:15 2011<br>0.00100g/mlMeOH<br>LZYD8-1	Na<br>589nm	2 sec<br>10 sec
No.4	5 (1/3)	Sp.Rot	47.0000	0.0047<br>0.0000	12.6<br>10.00<br>Cell	Mon Dec 26 09:28:46 2011<br>0.00100g/mlMeOH<br>LZYD8-1	Na<br>589nm	2 sec<br>10 sec +43.2222°
No.5	5 (2/3)	Sp.Rot	40.0000	0.0040<br>0.0000	12.6<br>10.00<br>Cell	Mon Dec 26 09:29:00 2011<br>0.00100g/mlMeOH<br>LZYD8-1	Na<br>589nm	2 sec<br>10 sec
No.6	5 (3/3)	Sp.Rot	41.0000	0.0041<br>0.0000	12.6<br>10.00<br>Cell	Mon Dec 26 09:29:13 2011<br>0.00100g/mlMeOH<br>LZYD8-1	Na<br>589nm	2 sec<br>10 sec
No.7	7 (1/3)	Sp.Rot	32.0000	0.0032<br>0.0000	12.6<br>10.00<br>Cell	Mon Dec 26 09:31:09 2011<br>0.00100g/mlMeOH<br>LZYD8-1	Na<br>589nm	2 sec<br>10 sec
No.8	7 (2/3)	Sp.Rot	42.0000	0.0042<br>0.0000	12.6<br>10.00<br>Cell	Mon Dec 26 09:31:23 2011<br>0.00100g/mlMeOH<br>LZYD8-1	Na<br>589nm	2 sec<br>10 sec
No.9	7 (3/3)	Sp.Rot	40.0000	0.0040<br>0.0000	12.6<br>10.00<br>Cell	Mon Dec 26 09:31:36 2011<br>0.00100g/mlMeOH<br>LZYD8-1	Na<br>589nm	2 sec<br>10 sec

Figure 50. Optical rotation measurement of **5**.

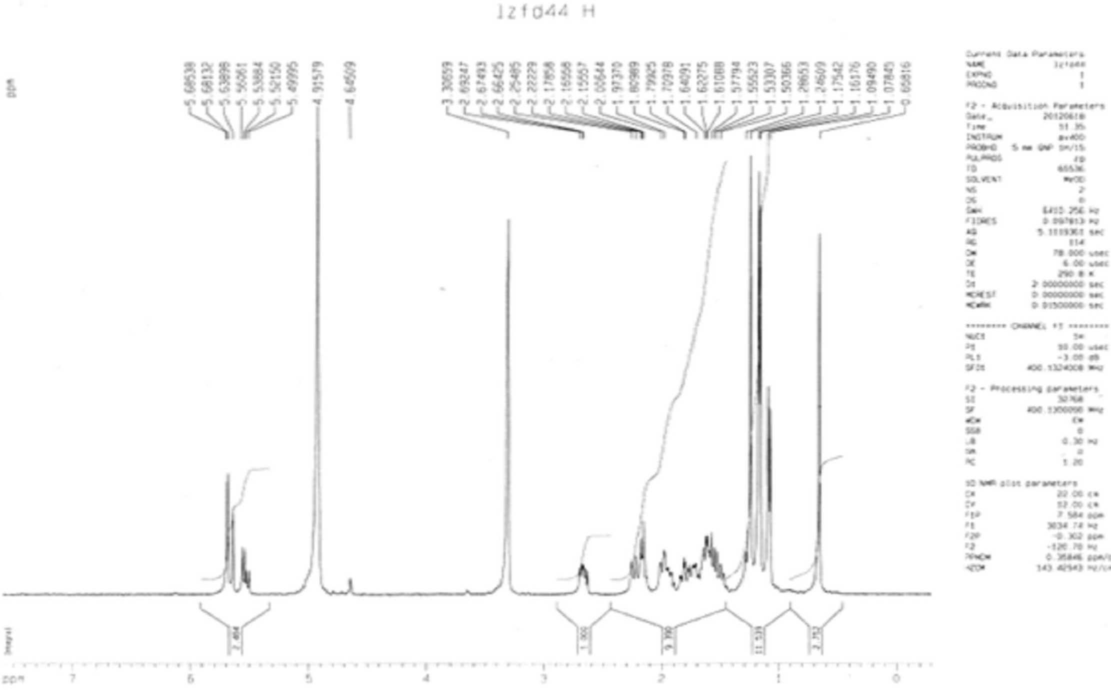

Figure 51. ^{1}H NMR spectrum of **6** (400 MHz, methanol-d_4).

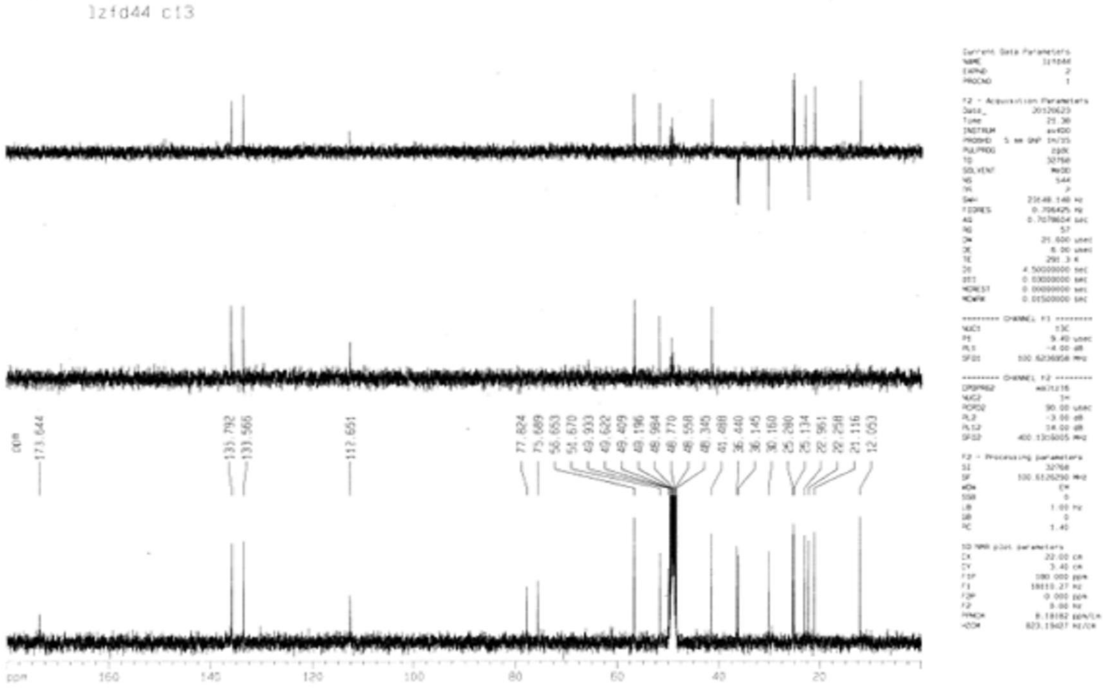

Figure 52. ^{13}C NMR spectrum of **6** (100 MHz, methanol-d_4).

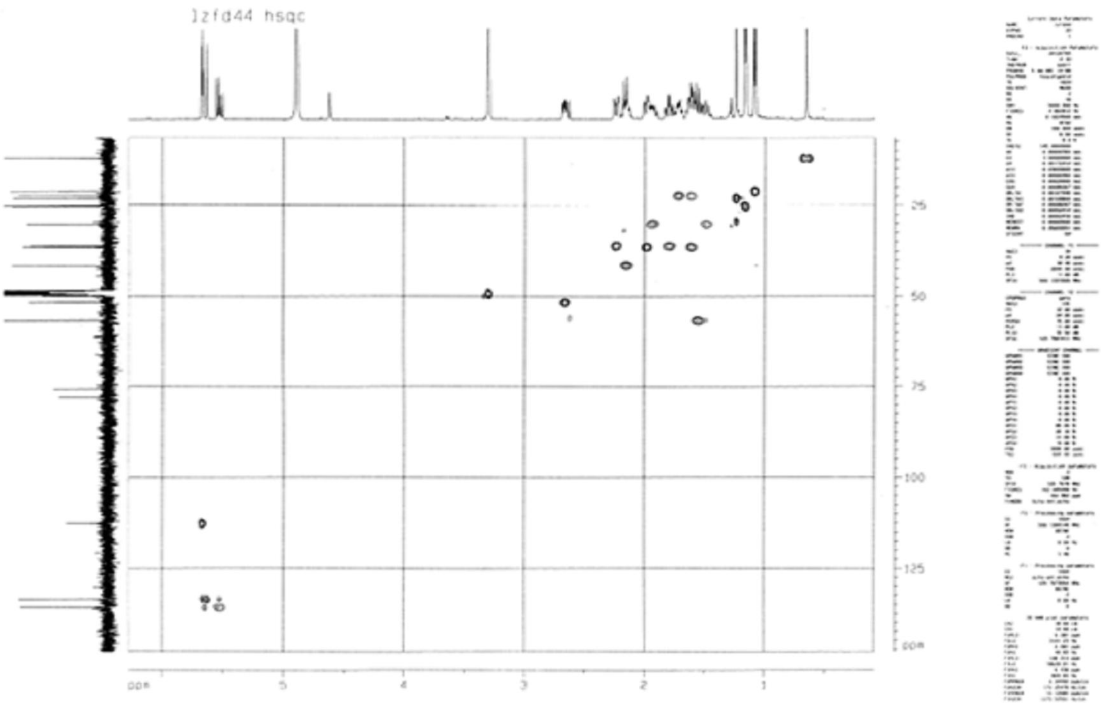

Figure 53. HSQC spectrum of **6** (500 MHz, methanol-d_4).

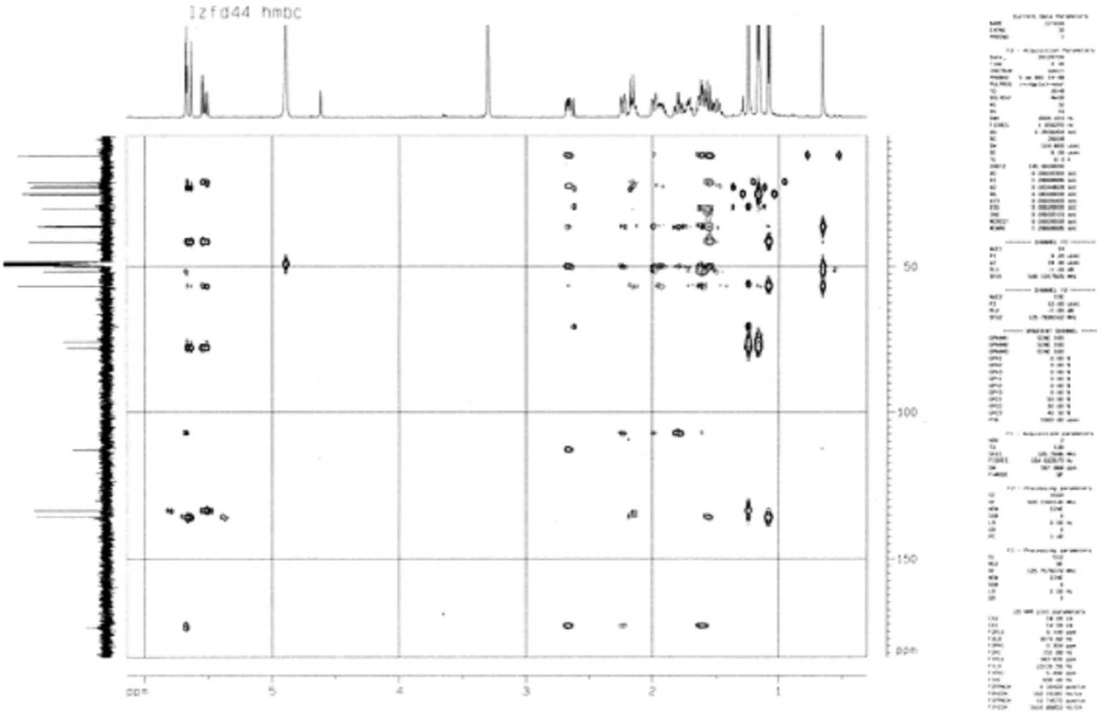

Figure 54. HMBC spectrum of **6** (500 MHz, methanol-d_4).

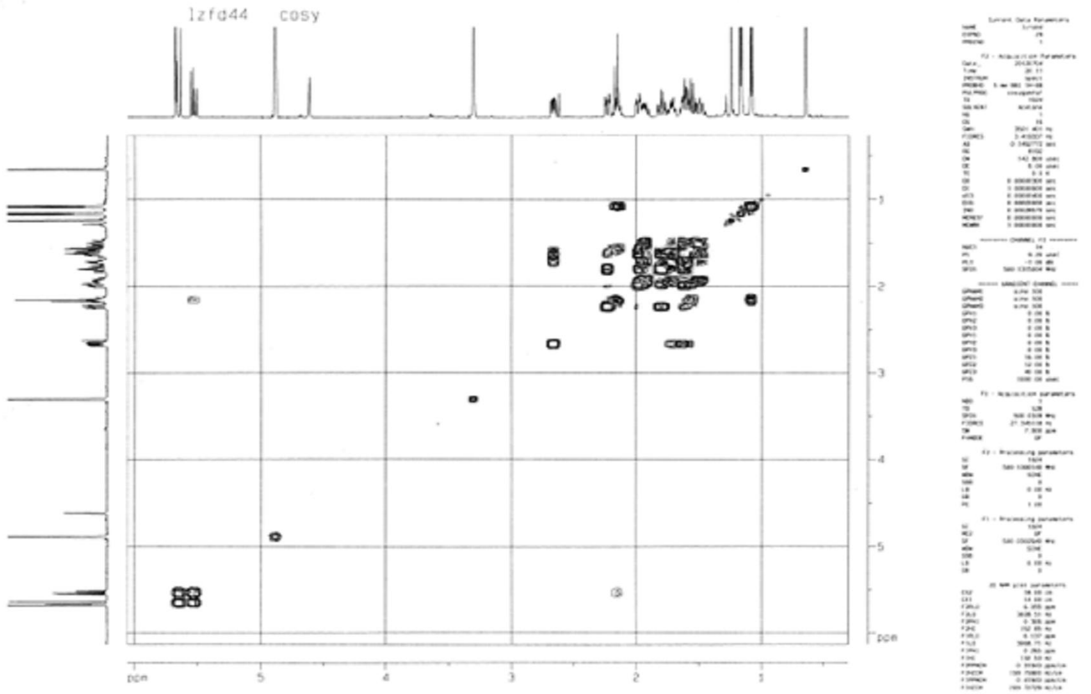

Figure 55. COSY spectrum of **6** (500 MHz, methanol-d_4).

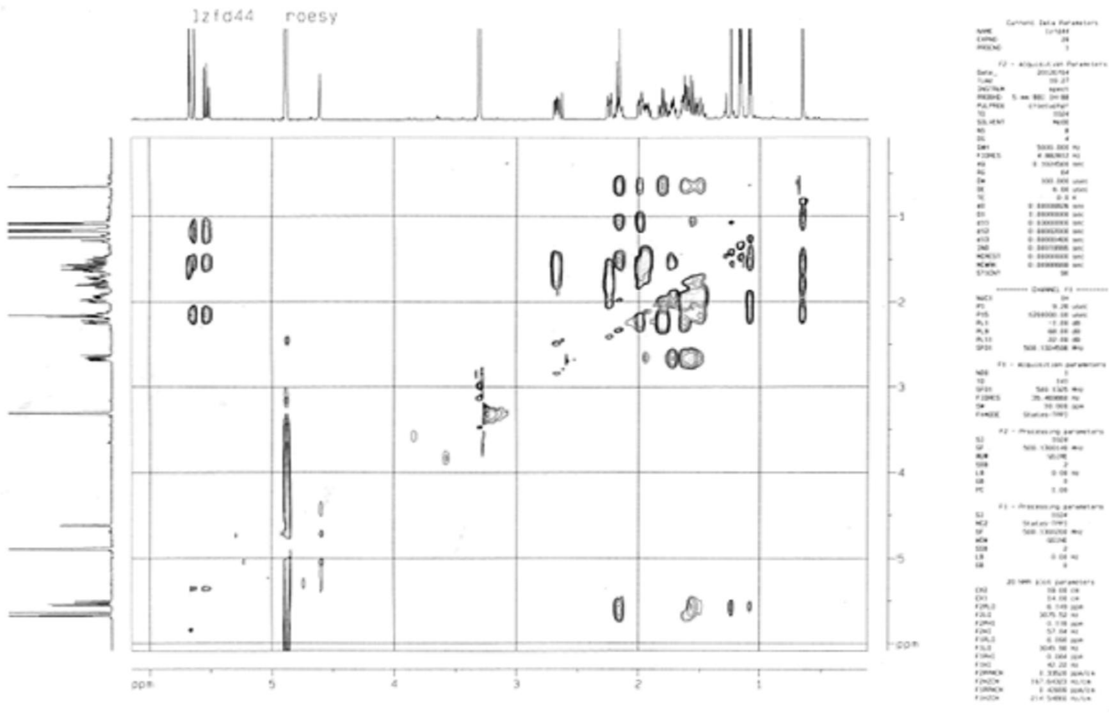

Figure 56. ROESY spectrum of **6** (500 MHz, methanol-d_4).

Elemental Composition Report Page 1

Single Mass Analysis
Tolerance = 10.0 PPM / DBE: min = -10.0, max = 120.0
Selected filters: None

Monoisotopic Mass, Odd and Even Electron Ions
22 formula(e) evaluated with 1 results within limits (up to 51 closest results for each mass)
Elements Used:
C: 0-200 H: 0-400 O: 3-6

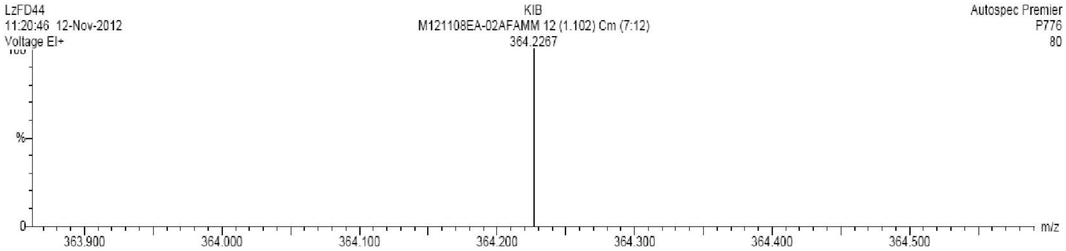

Minimum:				-10.0		
Maximum:		100.0	10.0	120.0		
Mass	Calc. Mass	mDa	PPM	DBE	i-FIT	Formula
364.2267	364.2250	1.7	4.7	6.0	5546054.5	C21 H32 O5

Figure 57. HR-ESI-MS of **6**.

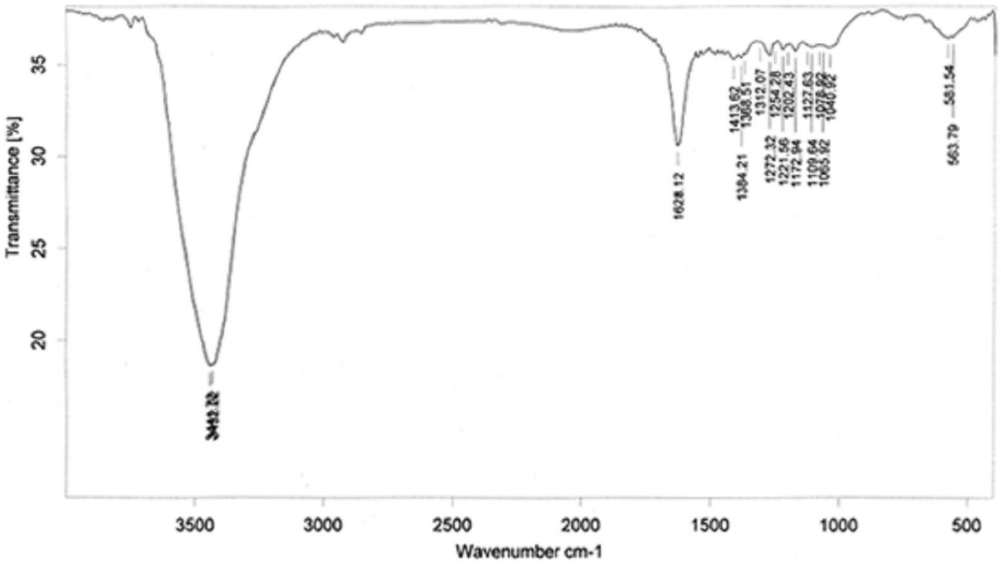

Sample : LzFD44	Frequency Range : 399.246 - 3996.32		Measured on : 31/12/2002
Technique : KBr压片	Resolution : 4	Instrument : Tensor27	Sample Scans : 16
Customer : 120927IR1	Zerofilling : 2	Acquisition : Double Sided,For	

Figure 58. IR spectrum of **6**.

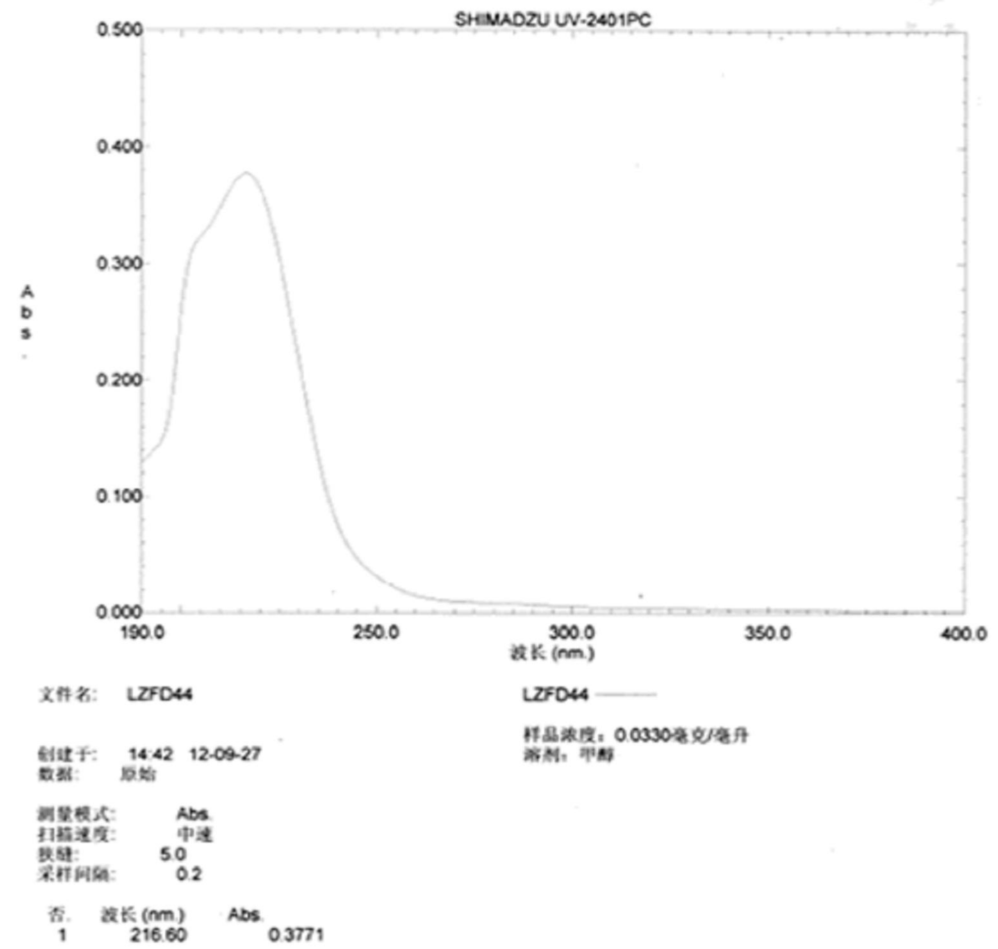

Figure 59. UV spectrum of **6**.

Optical rotation measurement

Model : P-1020 (A060460638)

No.	Sample	Mode	Data	Monitor<br>Blank	Temp.<br>Cell<br>Temp Point	Date<br>Comment<br>Sample Name	Light<br>Filter<br>Operator	Cycle Time<br>Integ Time
No.1	7 (1/3)	Sp.Rot	37.2730	0.0205<br>0.0000	21.3<br>50.00<br>Cell	Thu Sep 27 14:30:40 2012<br>0.00110g/mlMeOH<br>LZFD44	Na<br>589nm	2 sec<br>10 sec
No.2	7 (2/3)	Sp.Rot	33.4550	0.0184<br>0.0000	21.3<br>50.00<br>Cell	Thu Sep 27 14:30:53 2012<br>0.00110g/mlMeOH<br>LZFD44	Na<br>589nm	2 sec<br>10 sec
No.3	7 (3/3)	Sp.Rot	37.4550	0.0206<br>0.0000	21.3<br>50.00<br>Cell	Thu Sep 27 14:31:06 2012<br>0.00110g/mlMeOH<br>LZFD44	Na<br>589nm	2 sec<br>10 sec

+36.0606°

Figure 60. Optical rotation measurement of **6**.
